# Identification and Functional Validation of the Novel Antimalarial Resistance Locus *PF10_0355* in *Plasmodium falciparum*

**DOI:** 10.1371/journal.pgen.1001383

**Published:** 2011-04-21

**Authors:** Daria Van Tyne, Daniel J. Park, Stephen F. Schaffner, Daniel E. Neafsey, Elaine Angelino, Joseph F. Cortese, Kayla G. Barnes, David M. Rosen, Amanda K. Lukens, Rachel F. Daniels, Danny A. Milner, Charles A. Johnson, Ilya Shlyakhter, Sharon R. Grossman, Justin S. Becker, Daniel Yamins, Elinor K. Karlsson, Daouda Ndiaye, Ousmane Sarr, Souleymane Mboup, Christian Happi, Nicholas A. Furlotte, Eleazar Eskin, Hyun Min Kang, Daniel L. Hartl, Bruce W. Birren, Roger C. Wiegand, Eric S. Lander, Dyann F. Wirth, Sarah K. Volkman, Pardis C. Sabeti

**Affiliations:** 1 Department of Immunology and Infectious Diseases, Harvard School of Public Health, Boston, Massachusetts, United States of America; 2 Broad Institute, Cambridge, Massachusetts, United States of America; 3 Organismic and Evolutionary Biology, Harvard University, Cambridge, Massachusetts, United States of America; 4 FAS Center for Systems Biology, Harvard University, Cambridge, Massachusetts, United States of America; 5 Harvard Medical School, Boston, Massachusetts, United States of America; 6 Biological and Biomedical Sciences, Harvard University, Cambridge, Massachusetts, United States of America; 7 Faculty of Medicine and Pharmacy, Cheikh Anta Diop University, Dakar, Senegal; 8 Malaria Research Laboratories, College of Medicine, University of Ibadan, Ibadan, Nigeria; 9 Department of Computer Science and Department of Human Genetics, University of California Los Angeles, Los Angeles, California, United States of America; 10 Department of Biostatistics, University of Michigan, Ann Arbor, Michigan, United States of America; 11 School for Health Sciences, Simmons College, Boston, Massachusetts, United States of America; Yale University, United States of America

## Abstract

The *Plasmodium falciparum* parasite's ability to adapt to environmental pressures, such as the human immune system and antimalarial drugs, makes malaria an enduring burden to public health. Understanding the genetic basis of these adaptations is critical to intervening successfully against malaria. To that end, we created a high-density genotyping array that assays over 17,000 single nucleotide polymorphisms (∼1 SNP/kb), and applied it to 57 culture-adapted parasites from three continents. We characterized genome-wide genetic diversity within and between populations and identified numerous loci with signals of natural selection, suggesting their role in recent adaptation. In addition, we performed a genome-wide association study (GWAS), searching for loci correlated with resistance to thirteen antimalarials; we detected both known and novel resistance loci, including a new halofantrine resistance locus, *PF10_0355*. Through functional testing we demonstrated that *PF10_0355* overexpression decreases sensitivity to halofantrine, mefloquine, and lumefantrine, but not to structurally unrelated antimalarials, and that increased gene copy number mediates resistance. Our GWAS and follow-on functional validation demonstrate the potential of genome-wide studies to elucidate functionally important loci in the malaria parasite genome.

## Author Summary

Malaria infection with the human pathogen *Plasmodium falciparum* results in almost a million deaths each year, mostly in African children. Efforts to eliminate malaria are underway, but the parasite is adept at eluding both the human immune response and antimalarial treatments. Thus, it is important to understand how the parasite becomes resistant to drugs and to develop strategies to overcome resistance mechanisms. Toward this end, we used population genetic strategies to identify genetic loci that contribute to parasite adaptation and to identify candidate genes involved in drug resistance. We examined over 17,000 genetic variants across the parasite genome in over 50 strains in which we also measured responses to many known antimalarial compounds. We found a number of genetic loci showing signs of recent natural selection and a number of loci potentially involved in modulating the parasite's response to drugs. We further demonstrated that one of the novel candidate genes (*PF10_0355*) modulates resistance to the antimalarial compounds halofantrine, mefloquine, and lumefantrine. Overall, this study confirms that we can use genome-wide approaches to identify clinically relevant genes and demonstrates through functional testing that at least one of these candidate genes is indeed involved in antimalarial drug resistance.

## Introduction

*Plasmodium falciparum* malaria is a major public health challenge that contributes significantly to global morbidity and mortality. Efforts to control and eliminate malaria combine antimalarial drugs, bed nets and indoor residual spraying, with vaccine development a longer-term goal. Genetic variation in the parasite population threatens to undermine these efforts, as the parasite evolves rapidly to evade host immune systems, drugs and vaccines. Studying genetic variation in parasite populations will expand our understanding of basic parasite biology and its ability to adapt, and will allow us to track parasites as they respond to intervention efforts. Such understanding is increasingly important as countries move towards reducing disease burden and the ultimate elimination of malaria.

Given the potential impact of rapid evolution of *P. falciparum* in response to control and eradication strategies, discovery and characterization of *P. falciparum* genetic diversity has accelerated in recent years. Since the first malaria genome was sequenced in 2002 [1], over 60,000 unique SNPs have been identified by concerted sequencing efforts [2]–[4], and several genomic tiling arrays [5]–[9] and low-density SNP arrays [10], [11] have been developed to query this genetic variation. Recently the first malaria GWAS was published [11], in which 189 drug-phenotyped parasites from Asia, Africa and the Americas were genotyped using a low-density array (3,257 SNPs); that study identified loci under positive selection and found several novel drug resistance candidates.

For our study, we adopted two strategies for identifying genes involved in the malaria parasite's adaptive response: searching for signals of recent or ongoing natural selection, and searching for loci associated with one important clinical adaptation—resistance to antimalarial drugs. To make these searches possible, we began by sequencing 9 geographically diverse strains of *P. falciparum* to identify novel variation, thereby doubling the number of publicly available SNPs to 111,536 (all accessible at plasmodb.org), and used these SNPs to develop a high-density genotyping array assaying 17,582 validated markers. After characterizing linkage disequilibrium and population structure in our samples, we used the arrays to search for evidence of both ongoing balancing selection and recent positive selection, and to carry out a GWAS that sought loci associated with resistance to thirteen antimalarial agents. We then followed up one of the novel loci associated with drug resistance in order to verify that variation there was biologically involved in modulating drug response.

## Results

### Genetic Diversity

**Figure 1:**
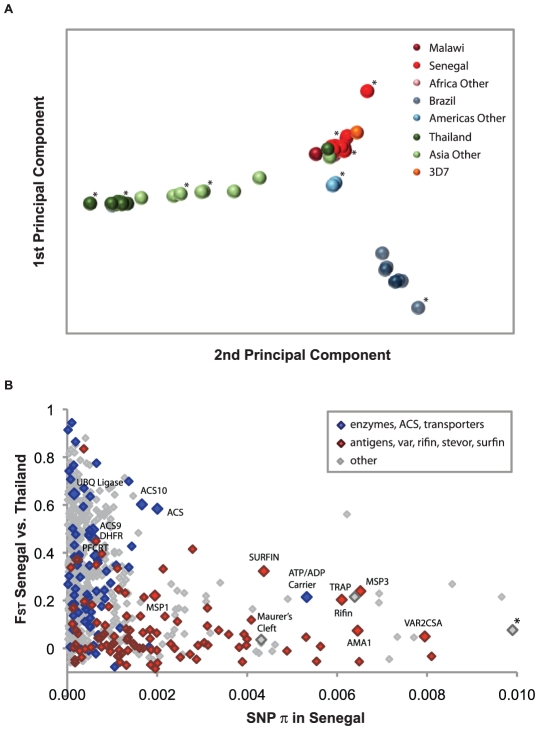
Parasite global population structure and genetic diversity versus divergence. (A) Population structure is visualized using the first two principal components of genetic variation for 57 parasites. Solid circles represent individual parasites, with colors assigned by reported origin: Africa in red, America in blue, and Asia in green. The nine strains used for ascertainment sequencing are indicated with (*). (B) Genetic diversity (SNP π) in Senegal versus divergence (F_ST_) between Senegal and Thailand is reported for 688 genes containing >3 successfully genotyped SNPs. Blue diamonds: enzymes, acyl-CoA synthetases (ACS) or transporters; red diamonds: antigens, vars, rifins, stevors or surfins; gray diamonds: all other genes. Gene IDs (PlasmoDB.org) for highlighted genes are listed in Table S7. A gene with unknown function is flagged with (*) to indicate that SNP π is off-scale (0.014).

We identified global population structure among malaria parasites using principal components analysis (PCA) of 57 genotyped culture-adapted parasite samples (Figure 1A, Table S1, Figure S1). African, American and Asian samples form three distinct clusters, reflecting the likely independent introduction of *P. falciparum* from Africa into Asia and the Americas. There was little evidence for structure within Africa, suggesting high gene flow throughout the region (Figure S1). Asian and American parasites however both show substantial internal structure.

There are also dramatic differences in linkage disequilibrium (LD) between populations, with substantial LD extending less than 1 kb in Senegal, 10 kb in Thailand, and 100 kb in Brazil (Figure S2). These observations are consistent with previous findings, which showed that LD decays more rapidly in Africa, due either to founder effects in other continents [12] or to elevated outcrossing frequencies in Africa [12], [13], where higher transmission intensity leads to a greater likelihood of sexual outcrossing rather than selfing within the mid-gut of vector mosquitoes.

The short LD in malaria, driven by high levels of recombination, means that a high density of markers is required to identify candidate loci in association studies, since causal variants not on the array can seldom be tagged by neighboring alleles (Table S2). On the other hand, short LD can aid in fine-mapping candidate associations and greatly accelerates the search for causal genes. Short LD also aids in identifying genomic regions under recent positive selection with recombination-based methods, since the increased LD in selected regions should stand out against the short-LD background.

### Detecting Signals of Natural Selection

We expect that many parasite proteins that interact with the host immune system will be under balancing selection, because they will be under selective pressure to maintain high levels of diversity. Indeed, previous studies have shown that regions of the *P. falciparum* genome that are highly polymorphic and appear to be under balancing selection encode antigens that are recognized by the human immune system [4]. We examined evidence for balancing selection in our data by searching for regions with high nucleotide diversity (as measured by SNP π) and low population divergence (as measured by F_ST_) (Figure 1B). When we examined the loci lying in this region of the graph (Figure S3), we found a number of known antigens and vaccine candidates. Loci in the same region with unknown function are thus potential novel antigens that trigger human immune response to malaria, and may prove useful as biomarkers or as candidate vaccine molecules.

We carried out a similar search for loci under positive selection by identifying regions with both low nucleotide diversity within Senegal and Thailand and high population divergence between the two populations (Figure 1B). We observed throughout the genome that nucleotide diversity was lower for nonsynonymous SNPs than for intergenic SNPs (Figure S4), a characteristic result of widespread purifying selection. At the same time, nonsynonymous SNPs exhibited significantly greater divergence than intergenic SNPs in all pairwise population comparisons, suggesting the effect of positive selection in local *P. falciparum* populations. Nonsynonymous SNPs with low diversity within a population and high divergence between the two populations studied may represent polymorphisms responsible for adaptive evolution.

We also carried out a genome-wide scan for recent positive selection using the long-range haplotype (LRH) test [14], which identifies common variants that have recently spread to high prevalence using recombination as a clock. Approximately 15 genes were identified as having undergone recent positive selection by this approach, including known drug resistance loci (*pfcrt* and *dhfr*) as well as multiple members of the acyl-CoA synthetase (ACS) and ubiquitin protein ligase families (Figures S5 and S6); these latter genes also exhibit high divergence between Senegal and Thailand (Figure 1B), evidence for selection that is recent and population-specific. Taken as a group, the genes identified by the LRH test show a significant enrichment for higher than average population divergence (as measured by F_ST_, Mann-Whitney U = 1583, P = 0.0071). All of these loci (Table S3, Dataset S1), which include genes in the folate metabolism, lipid biosynthesis and ubiquitin pathways, should be viewed as candidates for functional follow-up and further characterization.

### Genome-Wide Associations with Drug Resistance

In order to directly assess the genetic basis for one important response to antimalarial intervention, we carried out a GWAS to identify loci associated with drug resistance in parasites. This same approach can potentially be applied to many other clinically relevant malaria phenotypes, e.g. host response, invasion, and gametocyte formation. Our first step was to measure drug resistance (IC_50_ values) to 13 antimalarial drugs (amodiaquine, artemether, artesunate, artemisinin, atovaquone, chloroquine, dihydroartemisinin, halofuginone, halofantrine, lumefantrine, mefloquine, piperaquine and quinine) in 50 culture-adapted parasites using a high-throughput assay (Tables S4 and S5, Text S1, Dataset S1).

We performed the genome-wide association analysis using two statistical tests: efficient mixed-model association (EMMA) and a haplotype likelihood ratio (HLR) test (Figures S7, S8, S9, S10, Methods). EMMA identifies quantitative trait associations in individuals with complex population structure and hidden relatedness; it has previously been shown to outperform both PCA-based and λ_GC_-based correction approaches in highly inbred and structured mouse, maize, and Arabidopsis populations [15]. EMMA is particularly applicable for small and structured sample sets: one of its first applications was in a study of 24 diploid mouse strains [15], essentially the same sample size as in our study (50 haploid strains). The HLR test is a multi-marker test designed to detect the association of a single haplotype with a phenotype, and is particularly powerful when the associated haplotype experienced recent strong selection (and is therefore long) and occurs on a low-LD background [16]; it is therefore particularly appropriate for this study. We addressed the effect of population structure in the HLR test using population-specific permutation (Methods). When used together, these two complementary approaches provide a highly sensitive screen for association signals within the *P. falciparum* genome.

**Figure 2:**
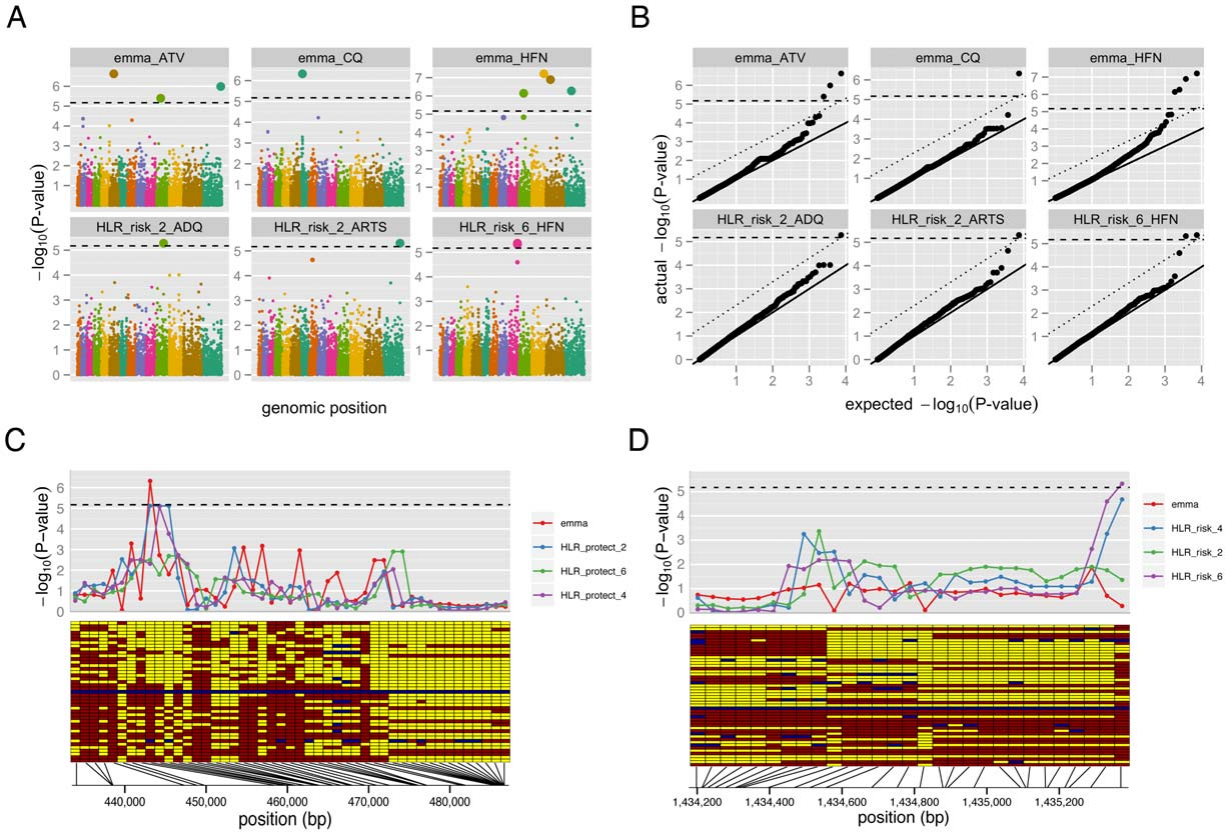
Genome-wide association study (GWAS) results. (A) Genome-wide significant associations were found for five antimalarials (out of thirteen tested) using EMMA and HLR tests. They include *pfcrt* (chromosome 7) associated with chloroquine resistance and eleven novel associations with resistance to several drugs, listed in Table 1. (B) Quantile-quantile plots for the *P*-value distributions in (A) show no significant confounding from population structure. Bonferroni-corrected genome-wide significance is marked with a dashed line; Benjamini-Hochberg significance is marked with a dotted line. (C-D) Close-ups are shown of the GWAS signal (top) and haplotypes (bottom) for resistance to (C) chloroquine (CQ) around the gene *pfcrt* and (D) halofantrine (HFN) around the gene *PF10_0355.* Yellow: sensitive allele; red: resistant allele; Blue: no data. Isolates are ordered by IC_50_, with the highest IC_50_ on the bottom.

The well-characterized chloroquine resistance locus, *pfcrt,* served as a positive control for our GWAS methods (Figure 2A and 2C, Table S2), an important test given our small sample size and the limited LD present in *P. falciparum*. As expected, we found evidence for association with resistance to chloroquine using both tests, consistent with previous studies [11]; EMMA yielded evidence for association with genome-wide signficance, while the signal from the HLR test fell just short of genome-wide significance (Figure 2C).

Applying the same tests to the other drug phenotypes, we detected numerous novel loci showing significant associations with drug resistance (Figure 2A and 2D, Table 1). Quantile-quantile plots for each test demonstrate that we were able to effectively control for population structure (Figure 2B). Despite our small sample size and the low LD in *P. falciparum*, in total eleven loci achieved genome-wide significance for association with resistance to five different drugs: amodiaquine, artemisinin, atovaquone, chloroquine and halofantrine. In most cases, the short extent of LD allowed localization to individual genes. Among the loci identified were various transporters and membrane proteins, as well as five conserved genes with unknown function (Table 1, Dataset S1).

**Table 1:** Eleven genome-wide significant associations with antimalarial drug resistance.

chr	SNPs	test	drug	*P*-value	genes	PlasmoDB description
6	674,154	EMMA	ATV	2.36E−07	PFF0785w	Ndc80 homologue, putative
7	459,787	EMMA	CQ	4.72E−07	MAL7P1_27	chloroquine resistance transporter
10	1,435,226, 1,435,286, 1,435,370, 1,437,695, 1,437,718, 1,441,590, 1,444,868	HLR_risk_6(2 overlapping hits)	HFN	4.71E−06, 4.25E−06	PF10_0355, PF10_0356	erythrocyte membrane protein putative, liver stage antigen 1
11	657,349	EMMA	ATV	4.01E−06	PF11_0178	conserved unknown
11	738,407	EMMA	HFN	7.20E−07	PF11_0203	peptidase, putative
11	1,123,028, 1,124,030	HLR_risk_2	ADQ	5.26E−06	PF11_0302	conserved unknown
12	1,964,935	EMMA	HFN	6.15E−08	PFL2285c	conserved unknown
13	757,689	EMMA	HFN	1.28E−07	PF13_0101	conserved unknown
14	1,233,470	EMMA	HFN	5.32E−07	PF14_0293	conserved unknown
14	2,814,793,2,815,714	HLR_risk_2	ARTS	4.90E−06	PF14_0654	aminophospholipid transporter, putative
14	3,130,449	EMMA	ATV	1.03E−06	PF14_0729	early transcribed membrane protein 14.2

Positions are given with respect to the PlasmoDB 5.0 reference assembly of 3D7. Drug abbreviations are ATV: atovaquone; CQ: chloroquine; HFN: halofantrine; ADQ: amodiaquine; ARTS: artemisinin. The HLR test for CQ-*pfcrt* association is just below the genome-wide significance threshold and is omitted here, but is shown in Figure 2C.

### Functional Validation of a Novel Resistance Candidate

Demonstrating that a signal of association actually reflects a causal molecular process requires functional testing and validation of the candidate locus, both because of concerns about power and reproducibility of genetic association tests, and because even a robust statistical correlation need not imply biological causation. To confirm the ability of GWAS to identify functionally relevant candidates, we investigated one of our association findings, *PF10_0355,* in greater depth. This gene contains multiple SNPs associated with halofantrine resistance (Figure 2D), and encodes a putative erythrocyte membrane protein (PlasmoDB.org) characterized by high genetic diversity.

**Figure 3:**
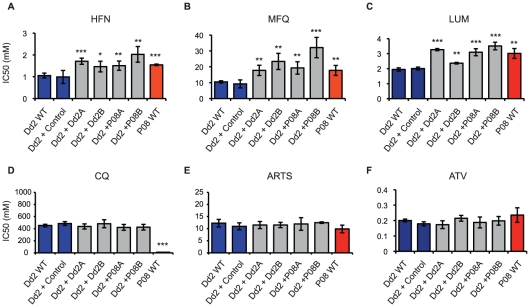
Overexpression of *PF10_0355* decreases parasite susceptibility to halofantrine (HFN) and related antimalarials. Parasite susceptibility to six antimalarials was measured by ^3^H-hypoxanthine incorporation. Comparisons were made between Dd2 (HFN-sensitive strain) and SenP08.04 (HFN-resistant strain), as well as 4 transfected lines. “Dd2+Dd2”: Dd2 parasites overexpressing *PF10_0355* from Dd2; “Dd2+P08”: Dd2 parasites overexpressing *PF10_0355* from SenP08.04. Overexpression of *PF10_0355* decreases parasite susceptibility to (A) HFN and structurally related (B) mefloquine (MFQ) and (C) lumefantrine (LUM). Overexpression of *PF10_0355* does not alter parasite susceptibility to (D) chloroquine (CQ), (E) artemisinin (ARTS) or (F) atovaquone (ATV). Mean IC_50_ ± standard error is shown. Significance levels: *: p<0.05, **: p<0.01, ***: p<0.001.

We set out to determine the role of *PF10_0355* in halofantrine resistance by transfecting halofantrine-sensitive Dd2 parasites with episomal plasmids containing the *PF10_0355* gene from a halofantrine-resistant parasite (SenP08.04), a technique that is used routinely for stable transgene expression [17]. Two independent transfectants overexpressing the *PF10_0355* gene from SenP08.04 both showed reduced susceptibility to halofantrine when compared with the Dd2 parent or a transfection control (Figure 3A), suggesting that this gene is indeed involved in modulating parasite drug response.

Two independent transfectants overexpressing the endogenous *PF10_0355* gene from halofantrine-sensitive Dd2 also showed reduced susceptibility to halofantrine (Figure 3A), however, pointing to a role of overexpression in the observed resistance. Because *PF10_0355* is annotated as a putative erythrocyte membrane protein and belongs to the merozoite surface protein 3/6 family, we tested the hypothesis that the observed effect was the by-product of a growth or invasion-related process, rather than resistance due to a direct interaction with the antimalarial itself. To that end, we expanded our drug testing in the transfectant lines to include other antimalarials, some structurally related and some unrelated to halofantrine.

**Figure 4:**
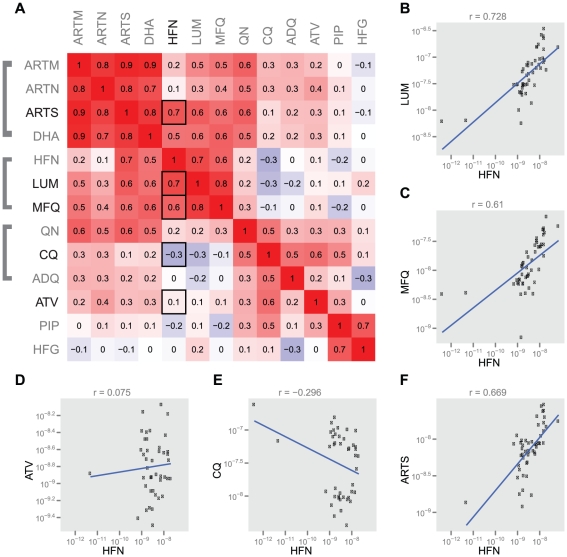
Correlations between antimalarial drugs tested. (A) Pearson correlation values (r) between log_10_(IC_50_) values are rendered as a color in a symmetric correlation matrix (red: correlated; white-uncorrelated, blue: inversely correlated). Thirteen antimalarials are measured: artemether (ARTM), artesunate (ARTN), artemisinin (ARTS), dihydroartemisinin (DHA), halofantrine (HFN), lumefantrine (LUM), mefloquine (MFQ), quinine (QN), chloroquine (CQ), amodiaquine (ADQ), atovaquone (ATV), piperaquine (PIP), and halofuginone (HFG). Drugs are grouped by structural relatedness. (B–F) Correlation plots are given with a linear regression line for HFN compared to the 5 other drugs tested for antimalarial resistance with *PF10_0355* overexpression: (B) LUM, (C) MFQ, (D) ATV, (E) CQ, and (F) ARTS.

Overexpression of *PF10_0355* from either the Dd2 or the SenP08.04 parent caused increased resistance to the structurally related antimalarials mefloquine and lumefantrine (Figure 3B and 3C), but had no effect on parasite susceptibility to the structurally unrelated antimalarials chloroquine, artemisinin or atovaquone (Figure 3D and 3E). Indeed, we found evidence of cross-resistance between halofantrine and both mefloquine and lumefantrine (Figure 4). We also observed cross-resistance between halofantrine and artemisinin, which is expected as cross-resistance between aminoquinolines and artemisinin compounds has been previously demonstrated [11], [18] and resistance to all these drugs has been shown to be mediated by changes in *pfmdr1* copy number [19], [20]. Overexpression of *PF10_0355*, however, alters parasite susceptibility to the aminoquinolines but not to artemisinin, suggesting that this effect is specific for that set of structurally related compounds and distinct from the effect of *pfmdr1*, which seems to exert a global effect of resistance to unrelated compounds (i.e. both aminoquinolines and artemisinins). Using the Dd2 parasite line, which has amplified *pfmdr1* copy number, as a background for *PF10_0355* overexpression allowed us to distinguish between cross-resistance to a structurally related class of compounds (mediated by *PF10_0355* overexpression) and pan-resistance to multiple classes of drugs.

**Figure 5:**
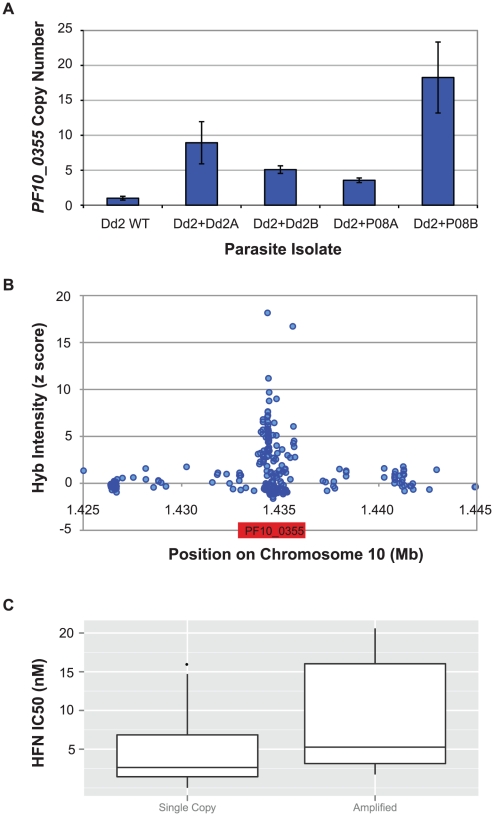
Copy number variation at *PF10_0355* is associated with HFN resistance. (A) Mean *PF10_0355* copy number (± standard deviation for three replicates) in the parent Dd2 and transfected lines from qPCR analysis. Dd2+Dd2: Dd2 parasites overexpressing *PF10_0355* from HFN-sensitive Dd2; Dd2+P08: Dd2 parasites overexpressing *PF10_0355* from HFN-resistant SenP08.04. Copy number was compared to the reference locus *PF07_0076*. (B) Increased hybridization intensity at *PF10_0355* on the high-density SNP array, measured by *Z*-scores for normalized and background-corrected data, for the HFN-resistant isolate SenP19.04. (C) Strains with increased copy number of *PF10_0355* (as measured by qPCR >1.2x 3D7) show a significantly higher resistance to HFN (p = 0.02, Student t-test).

Given that overexpression of the *PF10_0355* gene both from a halofantrine-resistant and from a sensitive parasite conferred resistance to halofantrine-related drugs, we investigated whether gene amplification might be driving the observed resistance, as it often does for antimalarial drugs [21]–[26]. We quantified *PF10_0355* copy number in our transfectants and found that the transfectant with the highest IC_50_ for all three drugs (Dd2+P08B) also had the highest *PF10_0355* copy number, as measured by quantitative PCR (qPCR) (Figure 5A). Furthermore, when we examined the *PF10_0355* gene on our SNP array, we detected a substantial increase in hybridization intensity at the *PF10_0355* locus compared to the genome average, suggesting that this gene is amplified in some parasites (Figure 5B). The amplified region appears only to contain the *PF10_0355* gene itself and not surrounding loci. We observed a similar pattern at *pfmdr1* on chromosome 5, where copy number variation is well established (Figure S11). Follow-up qPCR analysis of 38 parasite lines confirmed that parasites with amplified *PF10_0355* have a greater mean halofantrine IC_50_. (Figure 5C, Table S6, Dataset S1). Copy number variation was further confirmed in a number of parasites by quantitative Southern blotting (Figure S12).

## Discussion

In this study we used natural selection and genome-wide association methods to probe the genetic basis of adaptation in *P. falciparum*. These approaches are complementary: scanning for selected loci permits an unbiased search for unknown adaptive changes, but provides little information about the processes at work, while GWAS gives a focused look at one easily identified (and clinically critical) adaptive phenotype. Results from both approaches open up new avenues for study, as we seek to understand the biological significance of the findings.

The specifics of our strategy were designed to cope with two potential limitations in applying genome-wide population genetic approaches to malaria: small sample sizes, due to the difficulty in adapting parasites to culture and assessing drug and other phenotypes; and a lack of correlation (LD) between nearby variants in the parasite genome, which limits our ability to infer untyped SNPs from genotyped markers. The second limitation we addressed by developing a high-density genotyping array (based on new sequencing), to increase the fraction of genetic variation that we could directly interrogate, while the effect of the first was mitigated by the phenotype we targeted in our GWAS.

Drug resistance is a phenotype well-suited for GWAS because it is expected to be caused by common alleles of large effect at few genomic loci [27]. If this is the case, associations will be much easier to detect than in a typical human GWAS, in which the phenotype is caused by alleles at many loci that are either rare or of small effect. Additionally, the haploid nature of the intra-erythrocytic stage of *P. falciparum* further heightens GWAS power by eliminating the issue of allelic dominance. Finally, the increased LD caused by recent selection for drug resistance counteracts the loss of power that comes from short LD, small sample size, and the temporal and geographic stratification of the parasite population that we examined. Thus, despite the potential limitations, we were able to detect a known drug resistance locus (*pfcrt*), observed little *P*-value inflation in our GWAS data (Figures S8, S9, S10), and identified a number of genome-wide significant loci associated with drug resistance. Part of this success was likely due to specific tests we used to account for population structure.

Going beyond these statistical tests, we went on to functionally validate one of these loci, demonstrating that increased *PF10_0355* copy number confer resistance to three structurally related antimalarial drugs. This demonstrates the feasibility of coupling GWAS and functional testing in the malaria parasite for identifying and validating novel drug resistance loci and illustrates the power of GWAS to find functionally important alleles.

Comparing our results to the recent GWAS described by Mu, et al. [11], which was also directed at finding drug-resistance loci, we see that, beyond the well-known *pfcrt* locus, there was no overlap between the associations identified by each study. Differing sets of drugs tested and analytical methods explain much of the disagreement. Of the eleven candidate associations in Table 1, one (that with *pfcrt*) was found by both studies, eight were associations with drugs not assayed in Mu, et al. (atovaquone and halofantrine), and two were found only with a haplotype-based test, an approach not used by Mu, et al. Our candidate locus at *PF10_0355*, in fact, would not have been detectable in the Mu study because it was identified only by the multi-marker HLR test, because it involved an association with halofantrine, and because the Mu, et al. genotyping array lacked markers within 4 kb of the gene (plasmoDB.org).

Different parasite populations and marker sets probably explain many of the dihydroartemisinin, mefloquine and quinine associations identified by Mu, et al. but not seen in our data set. The studies used different parasite population sets—theirs was weighted toward southeast Asian strains, and ours toward African strains—and selection pressures and selected alleles can both vary between populations. Our smaller sample size also means that we might lack power to identify some associations accessible to Mu, et al. These difficulties are reflected in human GWAS studies as well, where the ability to replicate associations using multiple tests and in different sample sets has also been challenging to achieve [28].

Ultimately, the disparities in loci identified point to the role of population analysis as a tool for candidate gene discovery and not as a definitive study. Even within each study, there is little overlap between the signals observed with different methods—our study detects only one gene (*pfcrt*) by both GWAS tests (EMMA and HLR), while Mu, et al. detected only two genes (unknowns, not *pfcrt*) by both of their GWAS tests (Eigensoft and PLINK). Even a well-designed GWAS serves only as a hypothesis-generating experiment, and it is vital to empirically validate candidate loci associated with a phenotype of interest. Especially given the small sample sizes and relatively sparse marker density used in both malaria GWAS studies to date, functional validation of candidates is necessary to address concerns about false positive results.

Our functional result, that increased *PF10_0355* copy number confers decreased susceptibility to halofantrine, mefloquine and lumefantrine, raises additional questions for study. Further work will be needed to determine the precise contributions of copy number variation and gene mutation to the parasite's response to these drugs. The biological function of this gene's product is unknown, but previous work indicates putative localization to the parasite surface [29], as well as it being a potential target of host immunity and balancing selection [30]. While the protein itself does not appear to be a transporter, it is possible that it directly binds drug or perhaps couples with transport proteins to modulate drug susceptibility; interaction between membrane transporters and non-channel proteins has been demonstrated in cancer, plant and yeast systems [31]–[33]. Additional experiments are certainly required to determine the precise role of *PF10_0355* in modulating parasite response to this class of compounds, including assessing its relevance to resistance in natural populations, but it is clear that alteration of this locus can mediate drug resistance in *P. falciparum*.

Although halofantrine, mefloquine and lumefantrine are not commonly used as primary interventions, widespread halofantrine use has recently been documented in West Africa. Notably, halofantrine was used to treat nearly 18 million patients between 1988 and 2005 [34], [35], and it remains in production and use today. Use of halofantrine, mefloquine or lumefantrine as monotherapy may further explain how mutations and copy number variation in the *PF10_0355* gene were selected. Lumefantrine is also currently used as a partner drug in the artemisinin-based combination therapy (ACT) Coartem. The shorter half-life of artemether allows lumefantrine to be present as monotherapy, making it vulnerable to selection of drug resistant mutants. As genetic loci associated with drug responses are identified and validated, these provide new molecular biomarkers to evaluate drug use and response in malaria endemic settings. Thus, our findings have implications for defining molecular biomarkers for monitoring partner drug responses as intervention strategies, such as ACTs, are applied.

Beyond identifying a novel drug resistance locus, this study illustrates the general utility of a GWAS approach for the discovery of gene function in *P. falciparum*. Even with a small and geographically heterogeneous sample of parasites, we identified a number of new loci associated with drug response and validated one of them. Larger samples from a single population will have much greater power to detect additional loci, including those where multiple and low frequency alleles contribute to resistance. Future GWAS have the potential both to provide greater insights into basic parasite biology and to identify biomarkers for drug resistance and other clinically relevant phenotypes like acquired protection, pathogenesis, and placental malaria.

Future GWAS will be able to counteract the loss of power caused by low LD, either by focusing on parasite populations with reduced outcrossing rates, or by studying cases of very strong selective pressure. This issue will soon become moot, however, as the declining cost of whole-genome sequencing makes it practical to assay every nucleotide in the genome on a routine basis. Culture-adapted parasites are amenable to robust and reproducible phenotypic characterization, but their limitations—the potential for artifactual mutations during adaptation and for a biased selection of clones within a given infection—mean that genetic changes identified using them require both functional validation and demonstration that the changes are important during natural infection. As direct sequencing of clinical isolates with demonstrable clinical phenotypes such as *ex vivo* drug response or invasion properties becomes increasingly feasible, sequencing will enable us to directly identify genetic changes in the parasite associated with clinically relevant phenotypes. In the years ahead, genome analysis of *P. falciparum* has the potential to identify genetic loci associated with many phenotypes, enhance our understanding of the biology of this important human pathogen, and inform the development of diagnostic and surveillance tools for malaria eradication.

## Methods

### Parasites, Drug Testing, and DNA Isolation

Parasite samples and origins are detailed in Text S1 and Table S1. Parasites were maintained by standard methods [36] and were tested for their response to amodiaquine, artemether, artesunate, artemisinin, atovaquone, chloroquine, dihydroartemisinin, halofuginone, halofantrine, lumefantrine, mefloquine, piperaquine and quinine according to the methods outlined by Baniecki, et al. [37] (Table S4, Figure S13, Text S1). Follow-up drug testing was done by measuring uptake of ^3^H-hypoxanthine [38]. Nucleic acids were obtained from parasite cultures using Qiagen genomic-tips (Qiagen, USA). All DNA samples were evaluated by molecular barcode [39].

### Array Genotyping

We sequenced nine geographically diverse parasite isolates to 1.25x coverage, nearly doubling the number of publicly available SNPs to 111,536 (Text S1). These parasites had been previously sequenced to 0.25x coverage [2] and the deeper sequencing allowed for more thorough SNP discovery. Using this combined marker set, we created a high-density Affymetrix-based SNP array for *P. falciparum* containing 74,656 markers. Arrays were hybridized to 57 independent parasite samples (Table S1), including 17 previously sequenced strains used as a validation set. Genotype calls were produced using the BRLMM-P algorithm [40]. Markers that did not demonstrate perfect concordance between sequence and array data for the 17 strains were removed (Text S1). The remaining 17,582 SNPs constituted the high-confidence marker set used throughout this study (median marker spacing 444 bp, mean spacing 1,316 bp). All genomic positions and translation consequences are listed with respect to the PlasmoDB 5.0 assembly and annotation. SNP genotype data are publicly available on plasmodb.org (release 6.0, July 2009) and dbSNP (Build B134, May 2011), accessible by searching for submission batches Pf_0002 (sequencing of nine isolates) and Pf_0003 (genotyping of 57 isolates) from submitter BROAD-GENOMEBIO. Genotype data is also available as Dataset S2.

### Principal Component Analyses

Principal components analysis (PCA) was performed using the program SmartPCA [41]. All single-infection samples were used for the analysis in Figure 1. Samples that tightly clustered with the wrong continental population (A4, Malayan Camp and T2_C6) represented likely cases of contamination and were thus omitted from all other analyses.

### Diversity/Divergence Analysis

We measured diversity using a statistic we term ‘SNP π,’ which quantifies the average number of pair-wise differences among samples from a given population at assayed SNPs. Population divergence was measured using F_ST,_ calculated using the method of Hudson, et al. [42]. Statistical evaluation of the significance of differences in SNP π and F_ST_ among populations was performed using a bootstrapping approach, where the SNP set was re-sampled with replacement and each statistic recomputed 1000 times.

### Linkage Disequilibrium (LD) Analysis

The statistic r^2^ was calculated within each population for all pairs of SNPs sharing the same chromosome [43]; pairs were binned by distance and averaged within each bin. The level of LD between unlinked markers was estimated by calculating r^2^ between all pairs of SNPs on different chromosomes. To determine the bias caused by small sample size, the unlinked calculation was repeated, with the change that for each pair of SNPs, the genotype for one was taken from one strain while the genotype for the second was taken from another strain. This background value of r^2^ was calculated separately for the possible pairs of different strains and then averaged. Only single infections, as assessed by molecular barcode, were used.

### Long Range Haplotype (LRH) Analysis

Because of the small number of samples, LRH results for individual continental populations had a high level of variance. Thus, we pooled together samples from Africa (n = 26) and Asia (n = 18, excluding India), as suggested by our PCA analysis. SNPs included in the analysis had a minor allele frequency of at least 0.05 and a call rate of at least 0.8; missing genotypes were imputed using PHASE. LRH analysis was performed using Sweep. Each SNP defined two core alleles, one base pair in length. We calculated relative extended haplotype homozygosity (REHH) for each core allele, to its left and right [44], yielding up to four REHH scores per SNP locus. We standardized the REHH scores as a function of core allele frequency, defined on a discrete grid from 0.05 to 0.95 with even spaces of 0.025. This yielded a normally-distributed set of *Z*-scores for which we calculated corresponding *P*-values and *Q*-values.

### Genome-Wide Association Study (GWAS)

We performed a GWAS for drug resistance to thirteen antimalarials across 50 of our genotyped samples. 7,437 SNPs that had a minor allele count of five samples as well as an 80% call rate under every phenotype condition were used for GWAS. A Bonferroni significance threshold of –log_10_(*P*-value) >5.17 was used for all tests. See Text S1 for more details on GWAS methods.

The Efficient Mixed-Model Association (EMMA) test [15] models quantitative trait associations to a data set with complex population structure and hidden relatedness. It calculates a genotype similarity matrix instead of discrete categories and does not require *a priori* specification of populations. The resulting *P*-value distributions demonstrate little remaining effect from population structure (Figure S8) while retaining power to find a number of associations at genome-wide significance (Figure S8, Figure 2A, Table 1).

The Haplotype Likelihood Ratio (HLR) test [16] models the likelihood that a single, resistant haplotype rose to dominance while all other haplotypes proportionally decreased. PLINK [45] is used to produce sliding window haplotypes across the genome and calculate haplotype frequencies for input to the HLR test. We produced input for all 2-, 4- and 6-marker windows. The LOD scores generated by the HLR test were converted to empirical pointwise *P*-values by performing approximately 370,000 permutations of the null model for each test condition, allowing us to calculate empirical *P*-values up to a significance of 10^−5.6^. We preserved population-specific phenotype frequencies by permuting only within each of three populations defined by our PCA analysis (Table S1). Resulting *P*-value distributions fit expectations well for the vast majority of test conditions (Figures S9, S10) and the test demonstrates power to detect a number of loci at genome-wide significance (Figure 2A, Table 1).

### Copy Number Variation (CNV)

Copy number was assessed by evaluating the hybridization intensity at the *PF10_0355* locus on the high-density SNP array (Text S1). Follow-up analyses were done by quantitative real-time PCR (qPCR) of the *PF10_0355* locus using the Delta Delta Ct method [46]. *PF10_0355* was compared to the reference locus *PF07_0076* and 3D7 was used as a reference strain. A summary of *PF10_0355* copy number for all parasite strains tested is provided in Table S6. Select resistant strains that were found to have multiple copies of *PF10_0355* were further analyzed by quantitative Southern blotting and *PF10_0355* copy number was compared to the *dhps* gene from the 3D7 strain [47].

### PF10_0355 Overexpression

The full length ORF of *PF10_0355* was amplified from either the Dd2 (HFN sensitive) or SenP08.04 (HFN resistant) parasite isolate and cloned into the pBIC009 plasmid under the expression of the *Hsp86* promoter. Plasmid DNA was isolated, tranfected into the Dd2 parasite strain and stable transfectants were selected with 2.5 nM WR99210 [48]. Parasites from two independent experiments for each vector type (Dd2+Dd2 and Dd2+SenP08.04) were isolated and successful transfection was confirmed by plasmid rescue as well as episome-specific PCR and sequencing. Additionally, a vector control strain was made by transfecting Dd2 parasites with the pBIC009 plasmid containing the firefly luciferase gene (EC 1.13.12.7).

## Supporting Information

Dataset S1 Drug data, PF10_0355 copy number data, and top GWAS and LRH hits.
(0.06 MB XLS)

Dataset S2 Genotype data. Tab separated text file containing genotype data for 57 isolates across 17,582 SNPs. Additional information such as translation consequences (based on PlasmoDB v5.0 annotations) are also provided.
(3.29 MB TXT)

Figure S1 Principal components analysis of population structure within A) Africa B) the Americas, and C) Asia. Plots of the first two principal components using Eigenstrat [16] using the Affymetrix array. Each solid circle represents an individual, and the color is assigned according to the reported origin.
(0.56 MB DOC)

Figure S2 Linkage disequilibrium (LD), measured by r^2^, for each of the three population samples (Senegal, Thailand, Brazil). Plotted are r^2^ for linked markers (red lines) and for unlinked markers (blue lines), as well as the level of background LD expected because of small sample size (green lines).
(0.06 MB DOC)

Figure S3 Genes were classified by gene ontology (GO) functional categories and stratified by level of nucleotide diversity (π) as estimated by *Z*-scores. Select categories (highest five and lowest five categories along with categories in between that differ by incremental *Z*-scores) are shown. The majority of genes in GO categories for molecules found at the cell membrane have high levels of nucleotide diversity, while most of the genes classified into GO categories for conserved molecules lack nucleotide diversity.
(0.51 MB DOC)

Figure S4 SNP diversity and divergence by translation consequence. Diversity at assayed SNPs (SNP π) and Divergence between different populations as assayed by F_ST_, for different classes of SNPs: intergenic (4,263 SNPs), intronic (584 SNPs), synonymous (3,957 SNPs), and nonsynonymous (8,778 SNPs). Intronic SNPs have the widest error bars due to their relative sparseness on the array. Non-synonymous SNPs are generally among the least diverse and most differentiated class of SNPs.
(1.58 MB EPS)

Figure S5 Relative extended haplotype homozygosity (REHH) scores. Relative extended haplotype homozygosity (REHH) scores prior to any normalization, plotted for each core allele, (A) indexed by chromosome and position, and colored by chromosome, and (B) as a function of core allele frequency.
(0.61 MB DOC)

Figure S6 Long-range haplotype (LRH) analysis yields genome-wide significant candidates for recent positive selection. For each core allele, we calculated relative extended haplotype homozygosity (REHH), and from the set of all REHH scores we calculated a corresponding distribution of *Q*-values. We plotted -log_10_(*Q*-value), for all *Q*-values <1, for each core allele, indexed by chromosome and position, and colored by chromosome. The red dotted line corresponds to the typical *Q*-value significance threshold of 0.05. Gene annotations from PlasmoDB.org for some significant scores are labeled. For comparison, the well-known sweeps around drug resistance loci *pfcrt* and *dhfr* are labeled. This data is also shown in tabular form in Table S3.
(0.23 MB TIF)

Figure S7 GWAS *P*-value distributions for Fisher's exact test, permuted Fisher's exact test, and Cochran-Mantel-Haenszel (CMH) tests. Quantile-quantile plots (qq-plots) show log *P*-values for every SNP on the y axis against the null expectation on the x axis. Fisher's exact test results generally show *P*-value inflation due to confounding effects from population structure for many drugs ("Fish"). As such, no results from this test are reported. To account for population structure, permutations of the null distribution were performed while preserving phenotypic associations to three predefined population clusters ("Fishp"). CMH also performs a stratified association test given predefined population clusters ("CMH"). The permuted Fisher's test and CMH test results show appropriate correction for population structure, but show no hits at genome-wide significance to report.
(0.47 MB TIF)

Figure S8 GWAS results for the Efficient Mixed-Model Association (EMMA) test. QQ-plots show little to no confounding effect from population structure, with the possible exception of artesunate (ARTN). The significant ARTN result is not reported in Table 1 or Figure 2 for this reason. Manhattan plots depict the genomic location of significant hits, also reported in Table 1 and Figure 2.
(0.61 MB TIF)

Figure S9 GWAS *P*-value distributions for the Haplotype Likelihood Ratio (HLR) tests for association for drug resistance. Population-sensitive permutations of the null model were used to calculate *P*-values from LOD scores. Final distributions of *P*-values show little to no confounding effect from population structure for most tests. Exceptions include the 6-SNP artemether (HLR_risk_6_ARTM) test and the 4-SNP amodiaquine (HLR_risk_4_ADQ) test--these results are not reported in Table 1 or Figure 2. Manhattan plots for other tests that reached genome-wide significance are in Figure 2A.
(3.52 MB TIF)

Figure S10 GWAS *P*-value distributions for Haplotype Likelihood Ratio (HLR) tests for association for drug sensitivity. Population-sensitive permutations of the null model were used to calculate *P*-values from LOD scores. Final distributions of *P*-values show little to no confounding effect from population structure. Genome-wide significant hits include piperaquine (HLR_protect_4_PIP) on a haplotype that spans *PF07_0126*, *PF07_0127* and *MAL7P1_167* and amodiaquine (HLR_protect_4_ADQ) on a haplotype in *PFL1800w*. A chloroquine hit on *pfcrt* just misses genome-wide significance. These results are not reported in Table 1.
(3.51 MB TIF)

Figure S11 Intensity *Z*-score for the Affymetrix array across chromosome 5. The results illustrate that probes for many of the SNPs assayed within the *pfmdr1* (888-988 k) locus exhibit notably higher hybridization intensity values in Dd2 relative to the other parasites, with 13 assays exhibiting average intensities greater than 2 standard deviations higher than observed in other strains. This is consistent with the copy number variation reported in the *pfmdr1* locus, with 3–4 copies present in the Dd2 strain relative to a collection of other strains.
(0.35 MB DOC)

Figure S12 *PF10_0355* copy number variation measured by Southern blotting. Select parasite isolates were digested with AflIII, EcoRV and XbaI and fragments were detected using probes to portions of the *PF10_0355* and *dhps* genes. Primers used for making probes were: *dhps* F: 5′-GTG ATT GTG TGG ATC AGA AGA TGA ATA ATC-3′; R: 5′-GGA TTA GGT ATA ACA AAA GGA CCA GAG G-3′; *PF10_0355* F: 5′-GGG GAA AGC ATA TAA TAA TAC TAT AGA TGC-3′; R: 5′-CTT GGA GGA ACA AGA ACC CCC TTA TTA TCA-3′ Radioactivity was measured using a phosphorimager plate and quantified using Quantity One software (version 4.6.5). Halofantrine (HFN) response is listed as sensitive (S) or resistant (R) for each strain.
(0.07 MB DOC)

Figure S13 Drug resistance phenotype classification for sweep and GWAS analyses. IC_50_ data were collected for thirteen anti-malarial drugs against all genotyped parasite lines. Quantitative IC_50_s were converted into binary "sensitive" and "resistant" phenotypes at the cutoffs shown (see also Table S4). These binary phenotypes were only used for the Haplotype Likelihood Ratio (HLR) test. Drug abbreviations: amodiaquine (ADQ), artemether (ARTM), artesunate (ARTN), artemisinin (ARTS), atovaquone (ATV), chloroquine (CQ), dihydroartemisinin (DHA), halofuginone (HFG), halofantrine (HFN), lumefantrine (LUM), mefloquine (MFQ), piperaquine (PIP) and quinine (QN).
(1.44 MB EPS)

Table S1 63 parasites used in the study with the name (parasite), geographic origin (region, country), source, and molecular barcode [8], as well as which samples were included in SNP discovery (SEQ), population characterization (POP), long-range haplotype (LRH), and GWAS analyses. For GWAS, * indicates that the sample was used, but not included in any population cluster for stratified or permuted analyses. The human control sample and the ancestral *P. reichenowi* sample were not used in any analyses reported here.
(0.12 MB DOC)

Table S2 Analysis of the ability of SNPs on the array to act as a proxy for or. This ability is measured using the standard correlation metric r^2^. In our data set, 28% of SNPs in the Brazilian sample (which has the most LD) had a nearby SNP on the array in strong LD (r^2^>0.5) with it, while in the Senegal sample the proportion was only 16%. Most of the time, therefore, we will only be able to detect association with markers that have been directly typed. The exception is strong selective sweeps, which affect many markers within a region.
(0.18 MB DOC)

Table S3 Long Range Haplotype (LRH) hits. All REHH hits with *Q*-value <0.25.
(0.19 MB DOC)

Table S4 IC_50_ drug resistance phenotype data (nM). ND: No data.
(0.12 MB DOC)

Table S5 Parasites used in the GWAS. Parasites used, indicating their nucleotide and amino acid sequence for various positions (indicated by number) in the *dhfr*, *pfcrt*, and *pfmdr1* gene loci.
(0.24 MB DOC)

Table S6 *PF10_0355* copy number summary for 38 parasites tested by qPCR using the Delta Delta Ct method. Copy number (CN) was compared to the reference locus *PF07_0076* and 3D7 was used as a reference strain. A cut-off of 1.4 was used to define *PF10_0355* copy number greater than 1; parasites with greater than 1 copy of *PF10_0355* are shaded. Parasites are ranked by Halofantrine (HFN) IC_50_: HFN-sensitive parasites are indicated by an S and HFN-resistant parasites are indicated by an R.
(0.08 MB DOC)

Table S7 Annotation and GeneID Information for identified genes in Figure 1B.
(0.05 MB DOC)

Text S1 Supplemental methods.
(1.29 MB PDF)
