# Supplementary figures and images for: Identification and Functional Validation of the Novel Antimalarial Resistance Locus PF10_0355 in Plasmodium falciparum
Source: PLoS Genet. 2011 Apr 21;7(4):e1001383. doi: 10.1371/journal.pgen.1001383 (PMC3080868; doi:10.1371/journal.pgen.1001383)

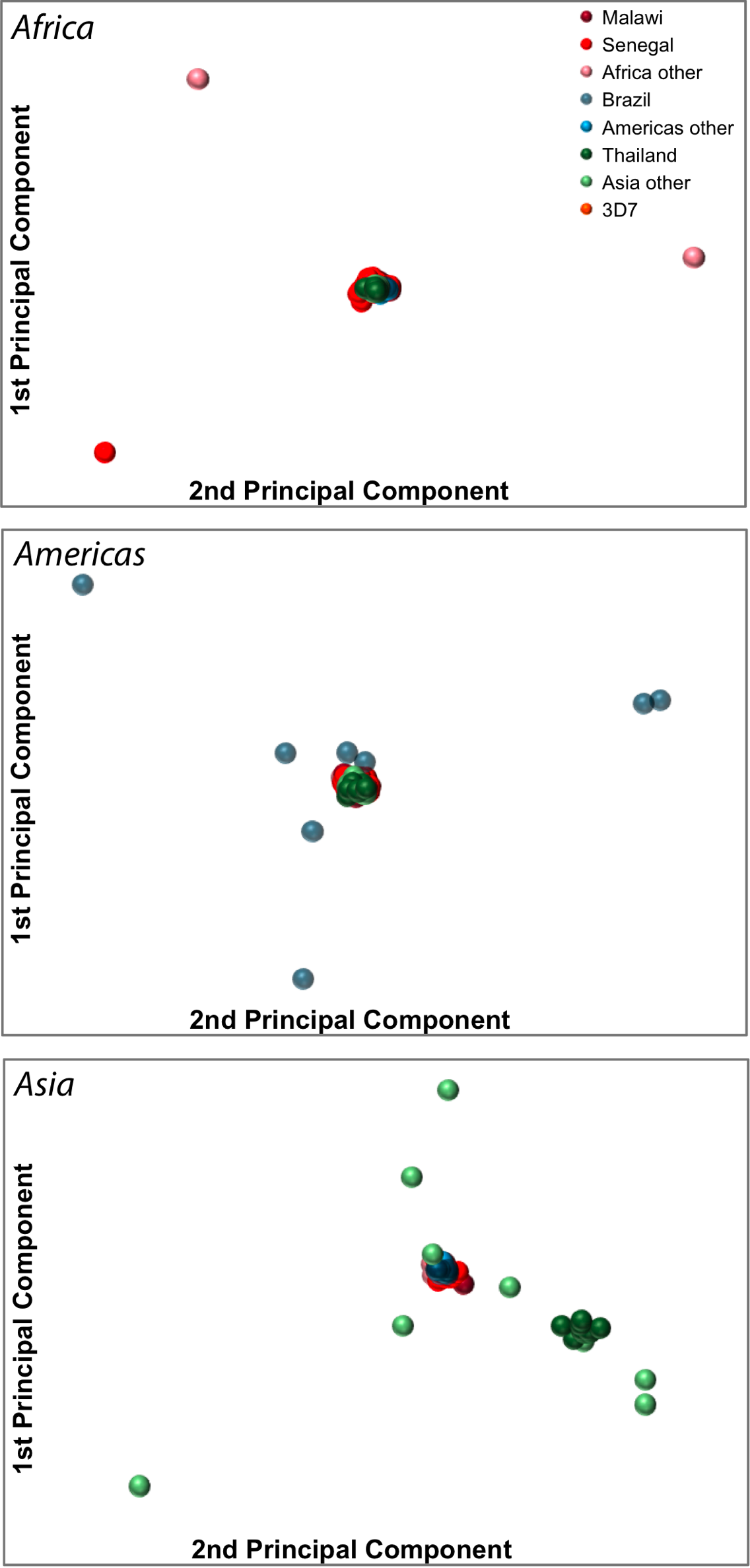

Supplement: Figure S1 — Principal components analysis of population structure within A) Africa B) the Americas, and C) Asia. Plots of the first two principal components using Eigenstrat [16] using the Affymetrix array. Each solid circle represents an individual, and the color is assigned according to the reported origin. (0.56 MB DOC) [file pgen.1001383.s003.doc]

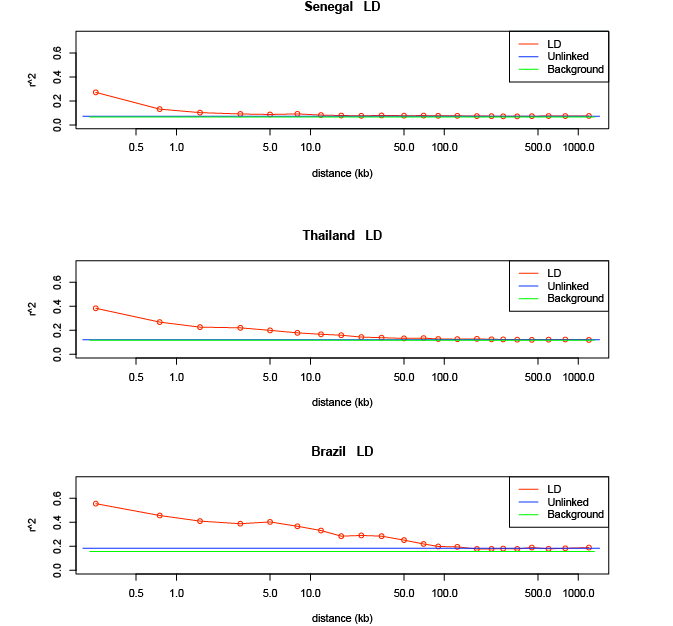

Supplement: Figure S2 — Linkage disequilibrium (LD), measured by r2, for each of the three population samples (Senegal, Thailand, Brazil). Plotted are r2 for linked markers (red lines) and for unlinked markers (blue lines), as well as the level of background LD expected because of small sample size (green lines). (0.06 MB DOC) [file pgen.1001383.s004.doc]

**
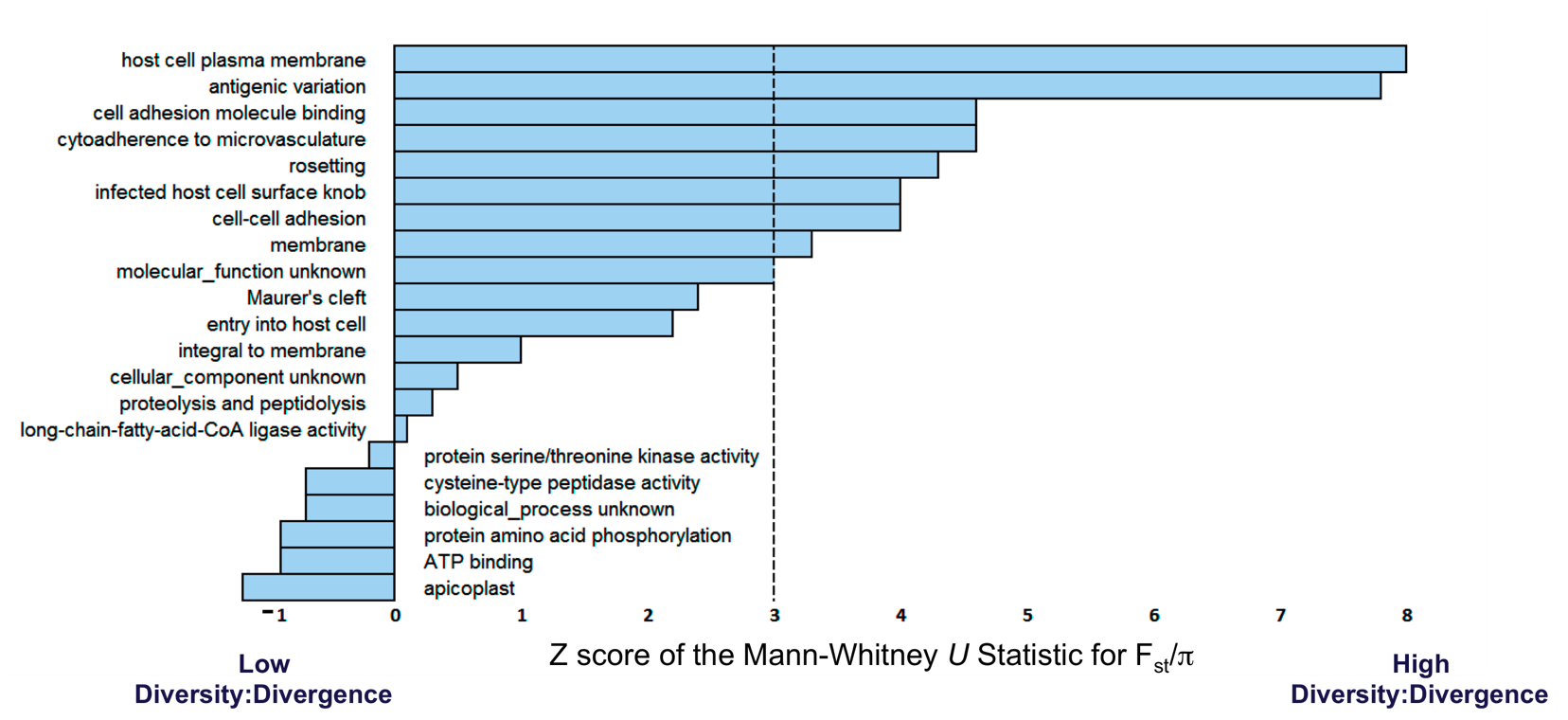
**

Supplement: Figure S3 — Genes were classified by gene ontology (GO) functional categories and stratified by level of nucleotide diversity (π) as estimated by Z-scores. Select categories (highest five and lowest five categories along with categories in between that differ by incremental Z-scores) are shown. The majority of genes in GO categories for molecules found at the cell membrane have high levels of nucleotide diversity, while most of the genes classified into GO categories for conserved molecules lack nucleotide diversity. (0.51 MB DOC) [file pgen.1001383.s005.doc]

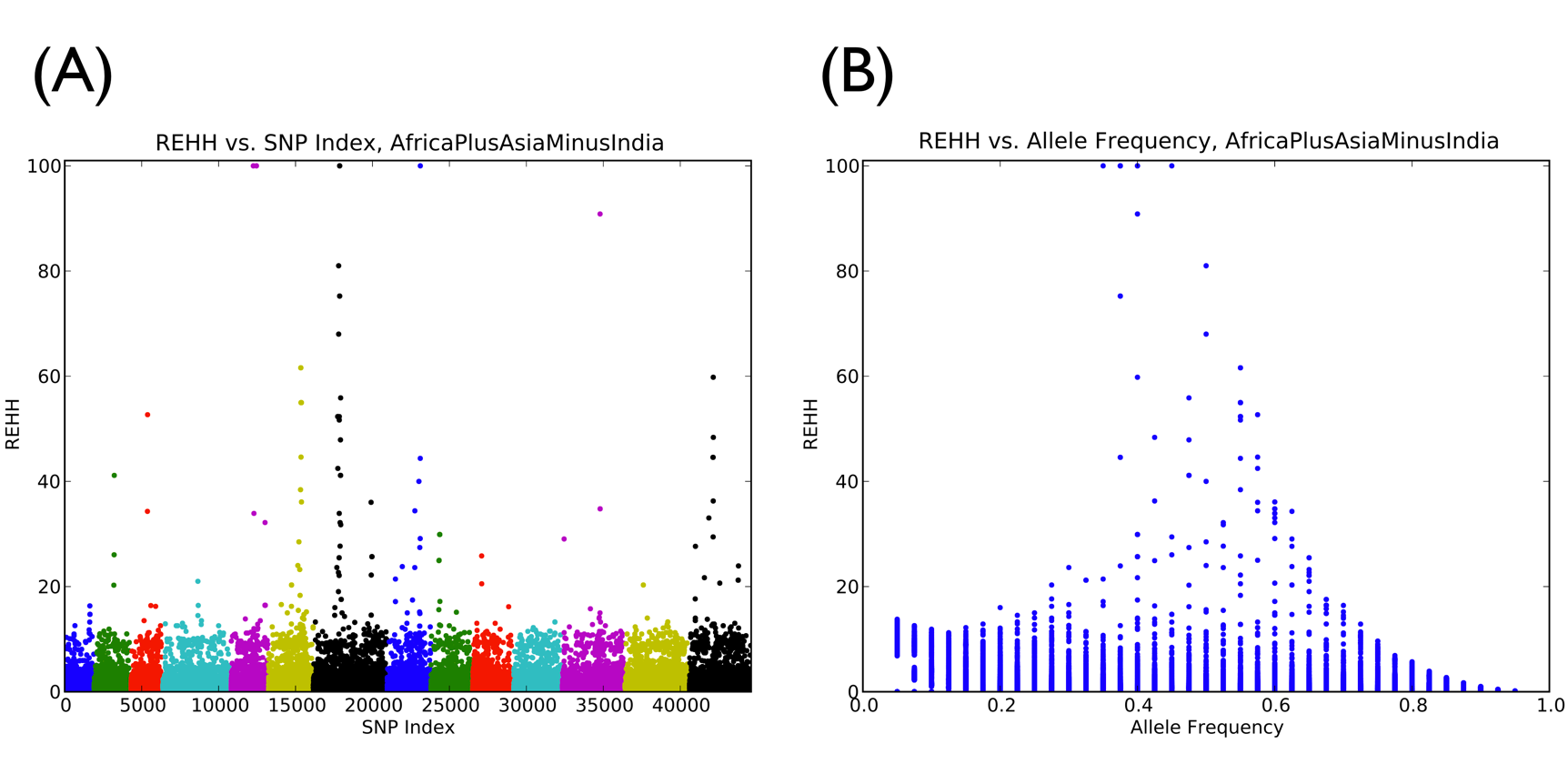

Supplement: Figure S5 — Relative extended haplotype homozygosity (REHH) scores. Relative extended haplotype homozygosity (REHH) scores prior to any normalization, plotted for each core allele, (A) indexed by chromosome and position, and colored by chromosome, and (B) as a function of core allele frequency. (0.61 MB DOC) [file pgen.1001383.s007.doc]

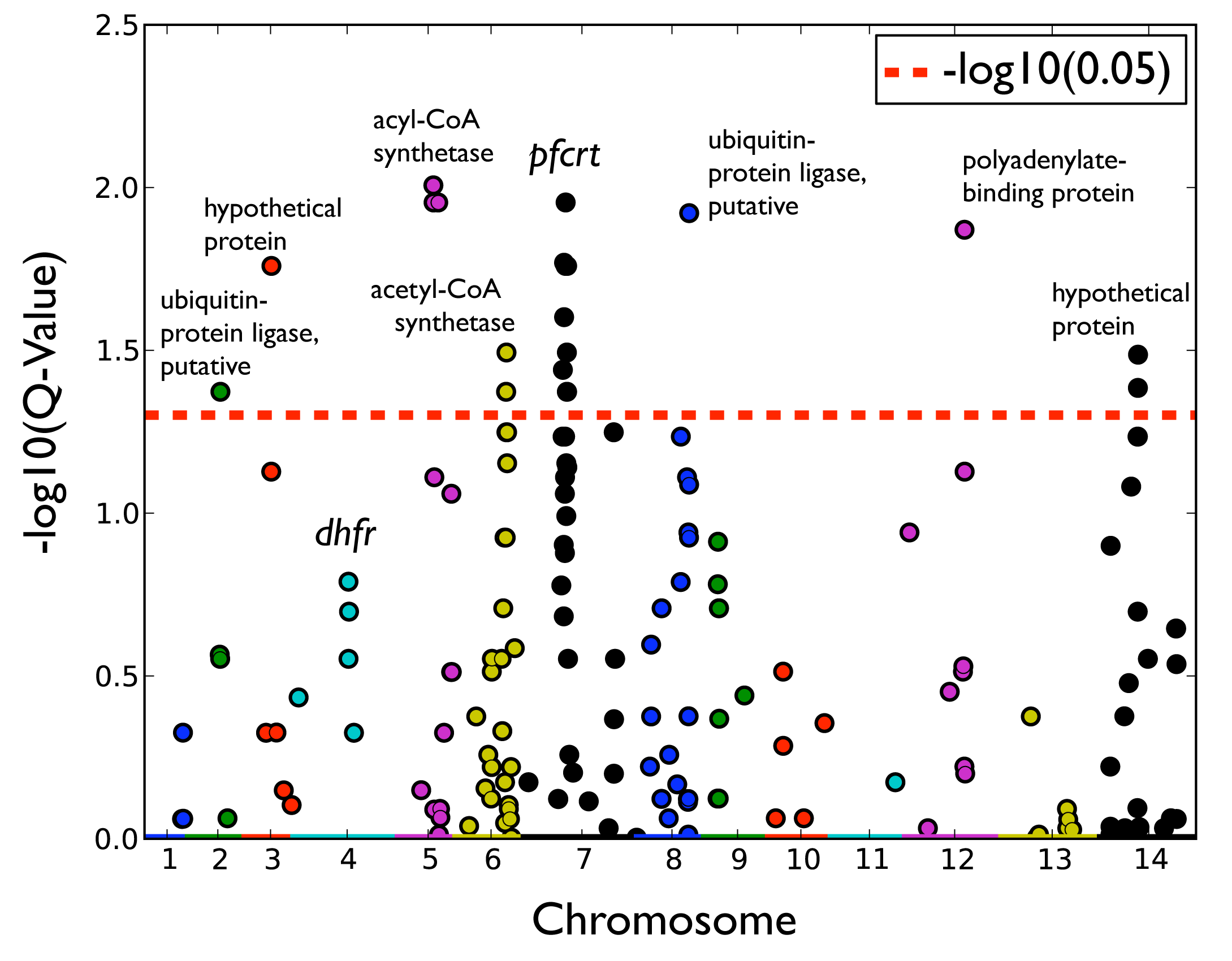

Supplement: Figure S6 — Long-range haplotype (LRH) analysis yields genome-wide significant candidates for recent positive selection. For each core allele, we calculated relative extended haplotype homozygosity (REHH), and from the set of all REHH scores we calculated a corresponding distribution of Q-values. We plotted -log10(Q-value), for all Q-values <1, for each core allele, indexed by chromosome and position, and colored by chromosome. The red dotted line corresponds to the typical Q-value significance threshold of 0.05. Gene annotations from PlasmoDB.org for some significant scores are labeled. For comparison, the well-known sweeps around drug resistance loci pfcrt and dhfr are labeled. This data is also shown in tabular form in Table S3. (0.23 MB TIF) [file pgen.1001383.s008.tif]

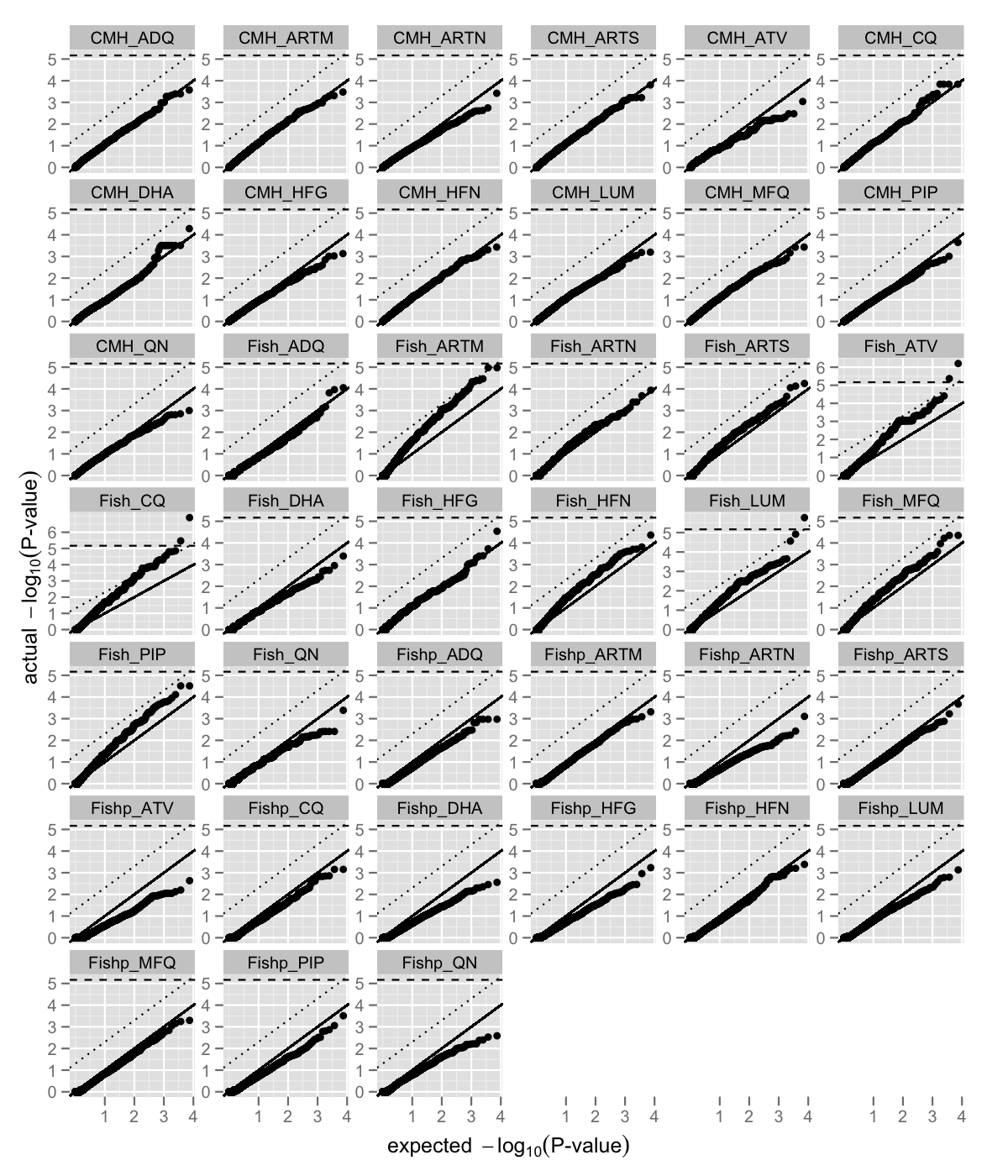

Supplement: Figure S7 — GWAS P-value distributions for Fisher's exact test, permuted Fisher's exact test, and Cochran-Mantel-Haenszel (CMH) tests. Quantile-quantile plots (qq-plots) show log P-values for every SNP on the y axis against the null expectation on the x axis. Fisher's exact test results generally show P-value inflation due to confounding effects from population structure for many drugs ("Fish"). As such, no results from this test are reported. To account for population structure, permutations of the null distribution were performed while preserving phenotypic associations to three predefined population clusters ("Fishp"). CMH also performs a stratified association test given predefined population clusters ("CMH"). The permuted Fisher's test and CMH test results show appropriate correction for population structure, but show no hits at genome-wide significance to report. (0.47 MB TIF) [file pgen.1001383.s009.tif]

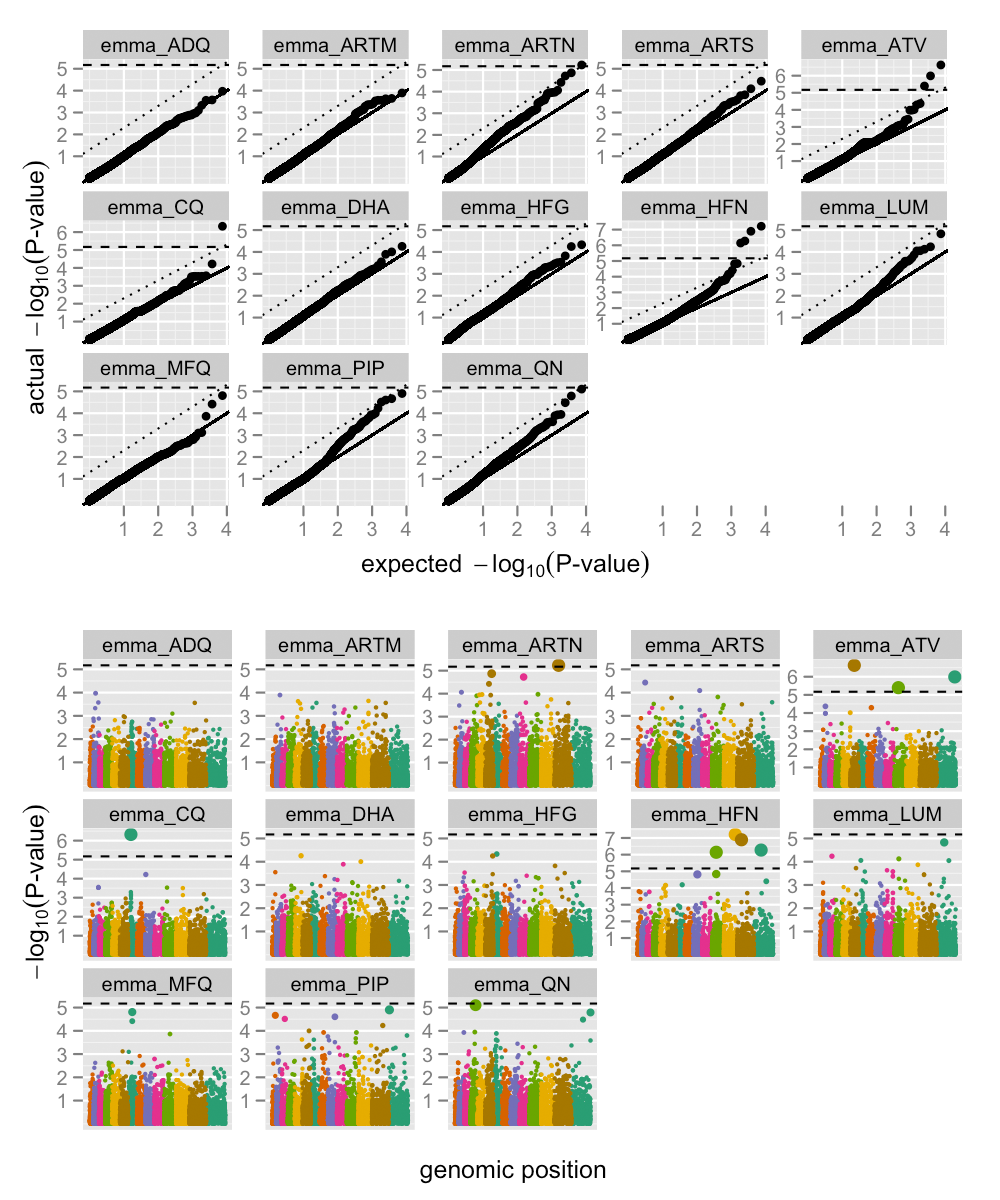

Supplement: Figure S8 — GWAS results for the Efficient Mixed-Model Association (EMMA) test. QQ-plots show little to no confounding effect from population structure, with the possible exception of artesunate (ARTN). The significant ARTN result is not reported in Table 1 or Figure 2 for this reason. Manhattan plots depict the genomic location of significant hits, also reported in Table 1 and Figure 2. (0.61 MB TIF) [file pgen.1001383.s010.tif]

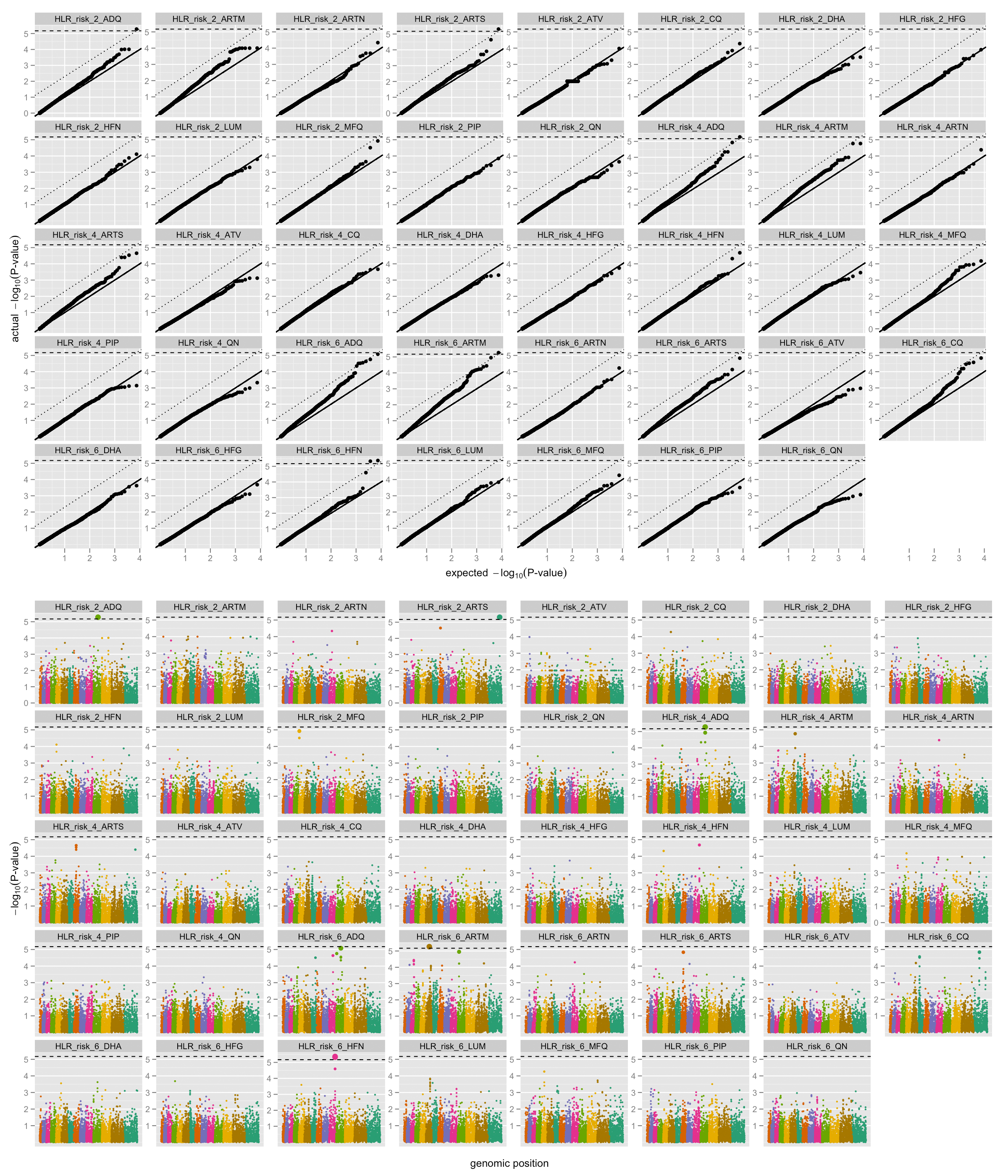

Supplement: Figure S9 — GWAS P-value distributions for the Haplotype Likelihood Ratio (HLR) tests for association for drug resistance. Population-sensitive permutations of the null model were used to calculate P-values from LOD scores. Final distributions of P-values show little to no confounding effect from population structure for most tests. Exceptions include the 6-SNP artemether (HLR_risk_6_ARTM) test and the 4-SNP amodiaquine (HLR_risk_4_ADQ) test--these results are not reported in Table 1 or Figure 2. Manhattan plots for other tests that reached genome-wide significance are in Figure 2A. (3.52 MB TIF) [file pgen.1001383.s011.tif]

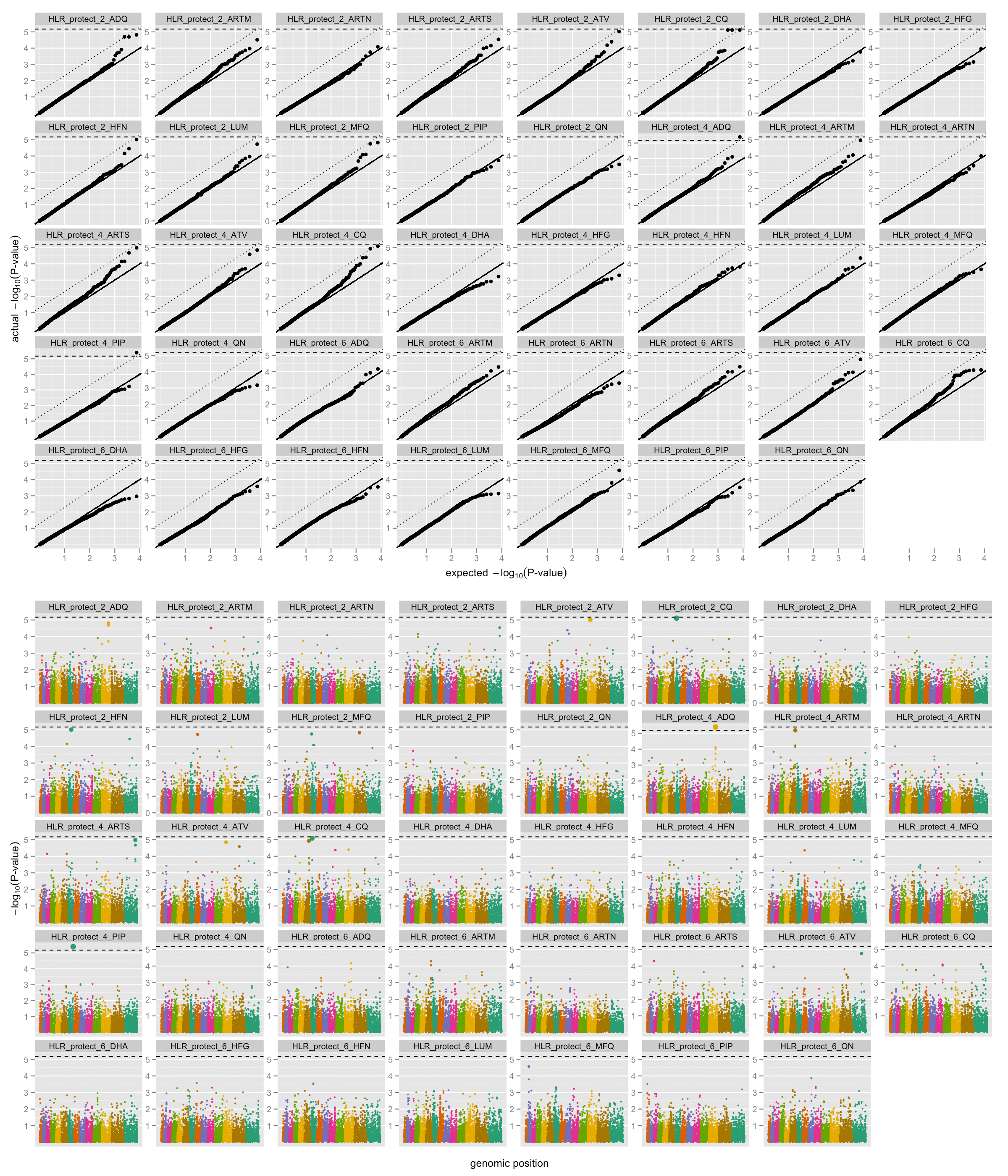

Supplement: Figure S10 — GWAS P-value distributions for Haplotype Likelihood Ratio (HLR) tests for association for drug sensitivity. Population-sensitive permutations of the null model were used to calculate P-values from LOD scores. Final distributions of P-values show little to no confounding effect from population structure. Genome-wide significant hits include piperaquine (HLR_protect_4_PIP) on a haplotype that spans PF07_0126, PF07_0127 and MAL7P1_167 and amodiaquine (HLR_protect_4_ADQ) on a haplotype in PFL1800w. A chloroquine hit on pfcrt just misses genome-wide significance. These results are not reported in Table 1. (3.51 MB TIF) [file pgen.1001383.s012.tif]

**
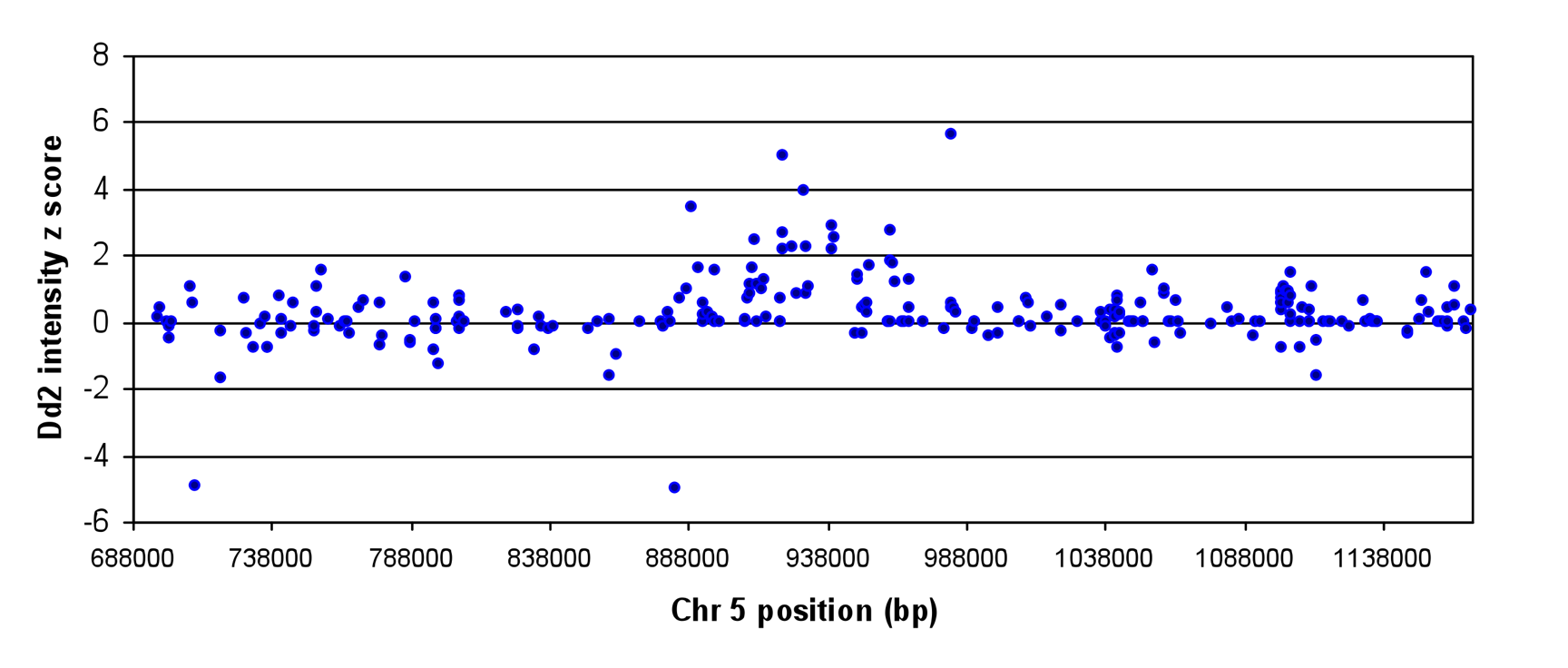
**

Supplement: Figure S11 — Intensity Z-score for the Affymetrix array across chromosome 5. The results illustrate that probes for many of the SNPs assayed within the pfmdr1 (888-988 k) locus exhibit notably higher hybridization intensity values in Dd2 relative to the other parasites, with 13 assays exhibiting average intensities greater than 2 standard deviations higher than observed in other strains. This is consistent with the copy number variation reported in the pfmdr1 locus, with 3–4 copies present in the Dd2 strain relative to a collection of other strains. (0.35 MB DOC) [file pgen.1001383.s013.doc]

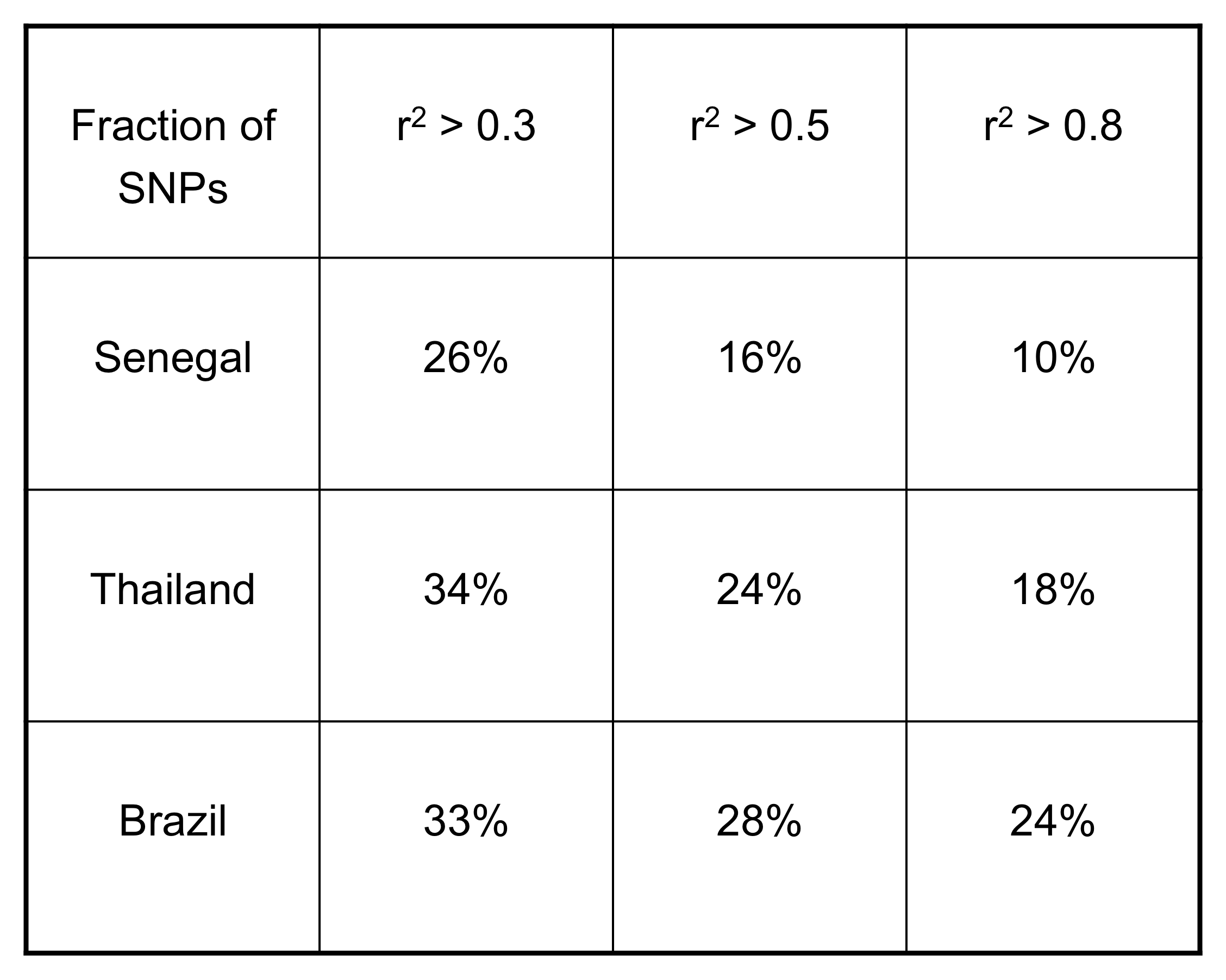

Supplement: Table S2 — Analysis of the ability of SNPs on the array to act as a proxy for or. This ability is measured using the standard correlation metric r2. In our data set, 28% of SNPs in the Brazilian sample (which has the most LD) had a nearby SNP on the array in strong LD (r2>0.5) with it, while in the Senegal sample the proportion was only 16%. Most of the time, therefore, we will only be able to detect association with markers that have been directly typed. The exception is strong selective sweeps, which affect many markers within a region. (0.18 MB DOC) [file pgen.1001383.s017.doc]
