# Supplementary material for: Identification and Functional Validation of the Novel Antimalarial Resistance Locus PF10_0355 in Plasmodium falciparum
Source: PLoS Genet. 2011 Apr 21;7(4):e1001383. doi: 10.1371/journal.pgen.1001383 (PMC3080868; doi:10.1371/journal.pgen.1001383)
Supplement: Table S1 — 63 parasites used in the study with the name (parasite), geographic origin (region, country), source, and molecular barcode [8], as well as which samples were included in SNP discovery (SEQ), population characterization (POP), long-range haplotype (LRH), and GWAS analyses. For GWAS, * indicates that the sample was used, but not included in any population cluster for stratified or permuted analyses. The human control sample and the ancestral P. reichenowi sample were not used in any analyses reported here. (0.12 MB DOC) [file pgen.1001383.s016.doc]

| ***Sample Information*** | | | | | ***Used in Analysis*** | | | |
| --- | --- | --- | --- | --- | --- | --- | --- | --- |
| ***Parasite*** | ***Region*** | ***Country*** | ***Source*** | ***Barcode*** | ***SEQ*** | ***POP*** | ***LRH*** | ***GWAS*** |
| 51 | America | Brazil | Alejandro Miguel Katzin | CATTGCAGACTXCACCTTAGATTG |  | x |  | x |
| 608 | America | Brazil | Alejandro Miguel Katzin | TACCCGGGATTACAAACTAGACTT |  | x |  | x |
| 10_54 | America | Brazil | Alejandro Miguel Katzin | CACTGCAGACTXTACACTAACCTG |  | x |  | x |
| 36_89 | America | Brazil | Alejandro Miguel Katzin | TACTGCAGATCGCCCCTACGCCTG |  | x |  | x |
| 365_89 | America | Brazil | Alejandro Miguel Katzin | TGCTCCGGATTACAAACAAGACTT |  | x |  |  |
| 3D7 | Europe | Netherlands | MRA-151 | TACTCCGGTCCGCACCCACGATGG |  | x | x | * |
| 7G8 | America | Brazil | MRA-152 | TACCCCAGACTXTCAATTAACCTG | x | x |  | x |
| 9_411 | America | Brazil | Alejandro Miguel Katzin | CACTCCAGACTGCAACTACGACTG |  | x |  | x |
| A4 | America | Brazil | J. Smith | TATTCCGGTTCATACCCXAGATTG |  | x | x |  |
| APO41 | Africa | Nigeria | Christian Happi | CATTGGGGTCTACACCCAAGACTG |  | x | x |  |
| CF04.008_12G | Africa | Malawi | Dan Milner | TACCCGGGACCGCCCACAAGATTG |  | x |  | x |
| CF04.008_1F | Africa | Malawi | Dan Milner | TATTCGGAACCGCACCCTAAATTG |  | x | x | x |
| CF04.009 | Africa | Malawi | Dan Milner | TATTCCGAACCGTACCCTCGATTG |  | x | x | x |
| D10 | Asia | PNG | MRA-201 | CACTCCAGATTGCAACTTAGCTTG | x | x | x | x |
| D6 | Africa | Sierra Leone | MRA-285 | TACTGGAAACTGCAACCAAACTTG | x | x | x | x |
| Dd2 | Asia | Indochina/Laos | MRA-156 | CATCGCAATTCGCCCCTTAGACTG |  | x | x | x |
| FCC2 | Asia | China | MRA-733 | TACCCCAAATCGCACATTAACCTG | x | x | x | x |
| GH2 | Asia | Thailand | S. Thaitong/D. Kyle | CACTGCGGTTTATCAATTAGCCTG |  | x | x | x |
| HB3 | America | Honduras | MRA-155 | TACTCCAGACTACACACTCACTTG |  | x |  | * |
| IGHCR14 | Asia | India | Aditya Dash/Chetan Chitnis | TACCCCAGACCXCACACTAGACGG |  | x |  | * |
| Indochina_I | Asia | Indochina/Laos | MRA-347 | TACTCGAGTCTACAACCACGATTG |  | x | x | x |
| JST | America | Brazil | Sandra do Lago Moraes | CACCGCGGTTTATAAACAAGATTG |  | x |  | x |
| K1 | Asia | Thailand | MRA-159 | TATTCGGATTTGTCCCTACGCCTG | x | x | x | x |
| M24 | Africa | Kenya | X. Su | CATTGCGGTTTACCCATAAGCCTG |  | x | x | x |
| Malayan Camp | Asia | Malaysia | MRA-330 | TATTCCGGATTGTCACTTAGACTG |  | x | x | * |
| Muz51.1 | Asia | PNG | Karen Day | TACTCCAGATTATCACCTAGCCTG |  | x | x | x |
| PR145 | Asia | Thailand | S. Thaitong/D. Kyle | CACTCCAGATCACAACCAAAACTG |  | x | x | x |
| PS189 | Africa | Mali | C. Plowe/Djimde | CACTCCGGATTACAAACAAGCTTT |  | x | x |  |
| RAJ116 | Asia | India | Aditya Dash/Chetan Chitnis | CACTCCGAACTGCAACCACAACGG |  | x |  | * |
| RO33 | Africa | Ghana | MRA-200 | CACCCGGGATCGCAAACTAAACTT | x | x | x | x |
| Santa Lucia | America | El Salvador | MRA-362 | CACCCGGGATTACAAACAAACCTT | x | x |  | * |
| SenP05.02 | Africa | Senegal | S. Mboup | CACCCGGGATTACAAACAAGCTTT |  | x | x | x |
| SenP08.04 | Africa | Senegal | S. Mboup | TACCCCGGATCGCAAACAAACTTT |  | x | x | x |
| SenP09.04 | Africa | Senegal | S. Mboup | CACTCGGGTTTATACATXCAACGT |  | x | x | x |
| SenP11.02 | Africa | Senegal | S. Mboup | CGCTCGAGATTACAAACTAGACTT |  | x | x | x |
| SenP19.04 | Africa | Senegal | S. Mboup | TGTTCCGGTCTACAAATTAACCGT |  | x | x | x |
| SenP26.04 | Africa | Senegal | S. Mboup | TATCCGAATTTATCAATACAACGT |  | x | x | x |
| SenP27.02 | Africa | Senegal | S. Mboup | TACTCCGGTTTATACACTCAACGT |  | x | x | x |
| SenP31.01 | Africa | Senegal | S. Mboup | TACTGCGGTCCGCAAACAAGATTG |  | x | x | x |
| SenP51.02 | Africa | Senegal | S. Mboup | TATCCGGGACTGCAACTTCGACGG |  | x | x | x |
| SenP60.02 | Africa | Senegal | S. Mboup | TACTCGAAACCGCAAACTAACCTT |  | x | x | x |
| SenT15.04 | Africa | Senegal | S. Mboup | TGCTCCAAATCGTACCCAAGCTGT |  | x | x | x |
| SenT26.04 | Africa | Senegal | S. Mboup | CGCTCGGATTTATCCCTACGCCGT |  | x | x | x |
| SenT28.04 | Africa | Senegal | S. Mboup | CACCCCAGTTTATCAACAAACTGT |  | x | x | x |
| SenV34.04 | Africa | Senegal | S. Mboup | TXCTCCAGATTATACCTAAACCTG | x | x | x | x |
| SenV35.04 | Africa | Senegal | S. Mboup | CGCTCGAGTCCGTCCACACACTGT |  | x | x | x |
| SenV42.05 | Africa | Senegal | S. Mboup | TGTTCCGATCTATCCACAAGACXT |  | x | x | x |
| T2_C6 | Asia | Thailand | MRA-818 | TACTCCGGATTATACACTAAACGT |  | x | x |  |
| TD203 | Asia | Thailand | S. Thaithong/D. Kyle | CGCCCCAGATCATAAATTAAACGT |  | x | x | x |
| TD257 | Asia | Thailand | S. Thaithong/D. Kyle | TACTCGGGATCGTAACCACACTGT |  | x | x | x |
| TM327 | Asia | Thailand | S. Thaithong/D. Kyle | TGTCCGAATCTATAAACACAACGT |  | x | x | x |
| TM345 | Asia | Thailand | S. Thaithong/D. Kyle | CACCCCAGTCTATCACTACACCGT |  | x | x |  |
| TM90C2A | Asia | Thailand | MRA-202 | TATTCCGATTTATCAACTCACCGT |  | x | x | x |
| TM90C6A | Asia | Thailand | MRA-205 | CACCCCAGTCTATCACTACACCGT |  | x | x |  |
| TM91C235 | Asia | Thailand | MRA-206 | CGCTCCGGACTGCACCCAAGATTG |  | x | x | x |
| V1/S | Asia | Vietnam | MRA-176 | TGCCCCAGATCACAACTAAGATTT | x | x | x | X |
| WR87 | Asia | Vietnam | MRA-284 | TACTGGAAATCACAACTAAGACTT |  | x | x | X |
| CF04.010 | Africa | Malawi | Dan Milner | mixed |  |  |  |  |
| Dd2_HFG_280 | Asia | Indochina/Laos |  |  |  |  |  |  |
| Human Control |  |  |  |  |  |  |  |  |
| Preichenowi |  |  | John Barnwell |  |  |  |  |  |
| SenT10.04 | Africa | Senegal | S. Mboup | NACTNGGGACTATAACCAAACCTG |  |  |  |  |
| TM93C1088 | Asia | Thailand | MRA-207 | CACCCCAGTCTATCACTACACCGT |  |  |  |  |
