# Supplementary material for: Identification and Functional Validation of the Novel Antimalarial Resistance Locus PF10_0355 in Plasmodium falciparum
Source: PLoS Genet. 2011 Apr 21;7(4):e1001383. doi: 10.1371/journal.pgen.1001383 (PMC3080868; doi:10.1371/journal.pgen.1001383)
Supplement: Table S3 — Long Range Haplotype (LRH) hits. All REHH hits with Q-value <0.25. (0.19 MB DOC) [file pgen.1001383.s018.doc]

| **chr** | **pos** | **core allele** | **hap len** | **qvalue** | **gene** | **description** |
| --- | --- | --- | --- | --- | --- | --- |
| 2 | 608790 | G | 14428 | 0.2071 | PFB0675w | hypothetical protein |
| 2 | 617743 | T | 16930 | 0.2133 | PFB0685c | acyl-CoA synthetase, PfACS9 |
| 2 | 623146 | C | 22333 | 0.0323 | PFB0687c | RING zinc finger protein, putative |
| 3 | 466483 | A | 35321 | 0.0568 | PFC0460w | hypothetical protein, conserved |
| 3 | 466610 | A | 35448 | 0.0133 | PFC0460w | hypothetical protein, conserved |
| 4 | 755220 | T | 15162 | 0.1236 | PFD0830w | bifunctional dihydrofolate reductase-thymidylate synthase |
| 4 | 755243 | C | 15139 | 0.2133 | PFD0830w | bifunctional dihydrofolate reductase-thymidylate synthase |
| 4 | 764100 | G | 50270 | 0.1528 | PFD0840w | hypothetical protein, conserved |
| 5 | 1042120 | A | 18768 | 0.0075 | PFE1250w | acetyl-CoA synthetase, PfACS10 |
| 5 | 1042527 | A | 18361 | 0.0085 | PFE1250w | acetyl-CoA synthetase, PfACS10 |
| 5 | 1056621 | T | 14545 | 0.0591 |  |  |
| 5 | 1159412 | C | 12844 | 0.0085 | PFE1400c | beta adaptin protein, putative |
| 5 | 1159501 | T | 12755 | 0.0085 | PFE1400c | beta adaptin protein, putative |
| 5 | 1333609 | G | 5132 | 0.2341 | PFE1640w | erythrocyte membrane protein 1 (PfEMP1), truncated |
| 5 | 1333639 | G | 5102 | 0.2341 | PFE1640w | erythrocyte membrane protein 1 (PfEMP1), truncated |
| 5 | 1333690 | T | 5051 | 0.0664 | PFE1640w | erythrocyte membrane protein 1 (PfEMP1), truncated |
| 5 | 1333703 | A | 5038 | 0.2341 | PFE1640w | erythrocyte membrane protein 1 (PfEMP1), truncated |
| 5 | 1333716 | G | 5025 | 0.2341 | PFE1640w | erythrocyte membrane protein 1 (PfEMP1), truncated |
| 5 | 1333729 | T | 5012 | 0.2341 | PFE1640w | erythrocyte membrane protein 1 (PfEMP1), truncated |
| 5 | 1333741 | G | 5000 | 0.2341 | PFE1640w | erythrocyte membrane protein 1 (PfEMP1), truncated |
| 5 | 1333790 | A | 4951 | 0.2341 | PFE1640w | erythrocyte membrane protein 1 (PfEMP1), truncated |
| 6 | 741192 | A | 55183 | 0.2335 |  |  |
| 6 | 741293 | C | 55082 | 0.2133 | PFF0855c | rifin |
| 6 | 741366 | A | 55009 | 0.2133 | PFF0855c | rifin |
| 6 | 1025852 | A | 17872 | 0.2133 | PFF1220w | hypothetical protein, conserved |
| 6 | 1065237 | G | 33215 | 0.1492 | PFF1280w | hypothetical protein, conserved |
| 6 | 1098314 | C | 26112 | 0.0905 | PFF1325c | c3h4-type ring finger protein, putative |
| 6 | 1114565 | C | 25158 | 0.0905 | PFF1350c | acetyl-coenzyme a synthetase |
| 6 | 1114929 | G | 23554 | 0.0323 | PFF1350c | acetyl-coenzyme a synthetase |
| 6 | 1115373 | A | 23998 | 0.0323 | PFF1350c | acetyl-coenzyme a synthetase |
| 6 | 1115454 | C | 23938 | 0.0430 | PFF1350c | acetyl-coenzyme a synthetase |
| 6 | 1116047 | G | 24531 | 0.0244 | PFF1350c | acetyl-coenzyme a synthetase |
| 6 | 1116171 | G | 24655 | 0.0430 | PFF1350c | acetyl-coenzyme a synthetase |
| 6 | 1116315 | C | 24799 | 0.0430 | PFF1350c | acetyl-coenzyme a synthetase |
| 6 | 1117520 | G | 26004 | 0.0430 | PFF1350c | acetyl-coenzyme a synthetase |
| 6 | 1124426 | C | 32910 | 0.0535 | PFF1365c | HECT-domain (ubiquitin-transferase), putative |
| 6 | 1283916 | G | 13004 | 0.1978 |  |  |
| 7 | 428373 | C | 39799 | 0.1269 | PF07_0027 | DNA-directed RNA polymerase 2 8.2 kDa polypeptide, putative |
| 7 | 449953 | C | 31672 | 0.0443 |  |  |
| 7 | 459787 | T | 21838 | 0.0276 | MAL7P1_27 | chloroquine resistance transporter |
| 7 | 460216 | G | 21409 | 0.0443 | MAL7P1_27 | chloroquine resistance transporter |
| 7 | 461218 | T | 20407 | 0.0443 | MAL7P1_27 | chloroquine resistance transporter |
| 7 | 465826 | G | 39654 | 0.0953 | PF07_0035 | cg1 protein |
| 7 | 465826 | G | 16307 | 0.1580 | PF07_0035 | cg1 protein |
| 7 | 467846 | G | 41674 | 0.0130 | PF07_0036 | Cg6 protein |
| 7 | 467846 | G | 14287 | 0.0190 | PF07_0036 | Cg6 protein |
| 7 | 475935 | T | 46758 | 0.0664 | PF07_0037 | Cg2 protein |
| 7 | 475935 | T | 26989 | 0.1009 | PF07_0037 | Cg2 protein |
| 7 | 475948 | A | 46771 | 0.0664 | PF07_0037 | Cg2 protein |
| 7 | 475948 | A | 26976 | 0.1009 | PF07_0037 | Cg2 protein |
| 7 | 476288 | G | 47111 | 0.0591 | PF07_0037 | Cg2 protein |
| 7 | 476305 | C | 47128 | 0.0443 | PF07_0037 | Cg2 protein |
| 7 | 476305 | C | 26619 | 0.0443 | PF07_0037 | Cg2 protein |
| 7 | 482133 | T | 10978 | 0.0133 | PF07_0038 | Cg7 protein |
| 7 | 482133 | T | 20791 | 0.0085 | PF07_0038 | Cg7 protein |
| **chr** | **pos** | **core allele** | **hap len** | **qvalue** | **gene** | **description** |
| 7 | 485744 | G | 14589 | 0.0133 | MAL7P1_28 | ribonucleases p/mrp protein subunit, putative |
| 7 | 485744 | G | 17180 | 0.0085 | MAL7P1_28 | ribonucleases p/mrp protein subunit, putative |
| 7 | 488164 | A | 15424 | 0.0777 | MAL7P1_28 | ribonucleases p/mrp protein subunit, putative |
| 7 | 488164 | A | 14760 | 0.0535 | MAL7P1_28 | ribonucleases p/mrp protein subunit, putative |
| 7 | 490748 | C | 18008 | 0.0323 | PF07_0040 | lysophospholipase-like protein, putative |
| 7 | 490748 | C | 14664 | 0.0244 | PF07_0040 | lysophospholipase-like protein, putative |
| 7 | 490877 | T | 18137 | 0.0323 | PF07_0040 | lysophospholipase-like protein, putative |
| 7 | 490877 | T | 14535 | 0.0133 | PF07_0040 | lysophospholipase-like protein, putative |
| 7 | 494285 | A | 21431 | 0.0551 | MAL7P1_29 | hypothetical protein, conserved |
| 7 | 505396 | G | 17088 | 0.2133 | MAL7P1_30 | hypothetical protein, conserved |
| 7 | 505412 | G | 17104 | 0.2133 | MAL7P1_30 | hypothetical protein, conserved |
| 7 | 936167 | A | 15183 | 0.0430 | MAL7P1_105 | hypothetical protein, conserved |
| 7 | 940007 | G | 11940 | 0.2133 | PF07_0085 | ferrodoxin reductase-like protein |
| 7 | 940111 | T | 12044 | 0.2133 | PF07_0085 | ferrodoxin reductase-like protein |
| 7 | 940147 | A | 12080 | 0.2133 | PF07_0085 | ferrodoxin reductase-like protein |
| 8 | 336524 | T | 6205 | 0.1930 | MAL8P1_135 | hypothetical membrane protein, conserved |
| 8 | 452794 | A | 778 | 0.1492 | PF08_0105 | rifin |
| 8 | 862485 | A | 33846 | 0.1239 | PF08_0054 | heat shock 70 kDa protein |
| 8 | 866334 | C | 29997 | 0.0443 | MAL8P1_64 | hypothetical protein, conserved |
| 8 | 1104023 | T | 8994 | 0.0591 |  |  |
| 8 | 1114567 | A | 8029 | 0.0873 | MAL8P1_23 | ubiquitin-protein ligase 1, putative |
| 8 | 1117372 | G | 10834 | 0.0905 | MAL8P1_23 | ubiquitin-protein ligase 1, putative |
| 8 | 1118090 | T | 11552 | 0.0622 | MAL8P1_23 | ubiquitin-protein ligase 1, putative |
| 8 | 1118190 | T | 11652 | 0.0091 | MAL8P1_23 | ubiquitin-protein ligase 1, putative |
| 9 | 272201 | C | 12632 | 0.1259 | PFI0265c | RhopH3 |
| 9 | 282410 | G | 11553 | 0.0932 | PFI0275w | hypothetical protein, conserved |
| 9 | 284833 | T | 12260 | 0.1492 | PFI0280c | autophagocytosis associated protein, putative |
| 9 | 284842 | T | 12269 | 0.1492 | PFI0280c | autophagocytosis associated protein, putative |
| 9 | 284910 | G | 12337 | 0.1492 | PFI0280c | autophagocytosis associated protein, putative |
| 10 | 324964 | G | 45666 | 0.2335 | PF10_0078 | histone deacetylase, putative |
| 12 | 50106 | T | 288 | 0.0873 | PFL0030c | erythrocyte membrane protein 1 (PfEMP1) |
| 12 | 947550 | A | 63271 | 0.2335 | PFL1130c | hypothetical protein, conserved |
| 12 | 954384 | C | 56437 | 0.2250 | PFL1130c | hypothetical protein, conserved |
| 12 | 990296 | T | 43303 | 0.0103 | PFL1170w | polyadenylate-binding protein, putative |
| 12 | 1002740 | T | 55747 | 0.0568 |  |  |
| 12 | 1002741 | A | 55748 | 0.0568 |  |  |
| 14 | 279667 | T | 34730 | 0.0959 | PF14_0074 | hypothetical protein |
| 14 | 960714 | G | 49486 | 0.0631 | PF14_0228 | hypothetical protein |
| 14 | 1225984 | A | 8890 | 0.0443 | PF14_0291 | hypothetical protein |
| 14 | 1226019 | T | 8925 | 0.0443 | PF14_0291 | hypothetical protein |
| 14 | 1226103 | C | 9009 | 0.1528 | PF14_0291 | hypothetical protein |
| 14 | 1226130 | C | 9036 | 0.0443 | PF14_0291 | hypothetical protein |
| 14 | 1226242 | A | 9148 | 0.0314 | PF14_0291 | hypothetical protein |
| 14 | 1226303 | T | 9209 | 0.0248 | PF14_0291 | hypothetical protein |
| 14 | 1608531 | A | 54272 | 0.2133 | PF14_0374 | hypothetical protein |
| 14 | 2812662 | C | 35145 | 0.1722 | PF14_0653 | hypothetical protein |
| 14 | 2812679 | T | 35128 | 0.1722 | PF14_0653 | hypothetical protein |
| 14 | 2838163 | G | 46456 | 0.2215 | PF14_0660 | hypothetical protein |
