# Supplementary material for: Identification and Functional Validation of the Novel Antimalarial Resistance Locus PF10_0355 in Plasmodium falciparum
Source: PLoS Genet. 2011 Apr 21;7(4):e1001383. doi: 10.1371/journal.pgen.1001383 (PMC3080868; doi:10.1371/journal.pgen.1001383)
Supplement: Table S4 — IC50 drug resistance phenotype data (nM). ND: No data. (0.12 MB DOC) [file pgen.1001383.s019.doc]

| **sample** | **ADQ** | **ARTM** | **ARTN** | **ARTS** | **ATV** | **CQ** | **DHA** | **HFG** | **HFN** | **LUM** | **MFQ** | **PIP** | **QN** |
| --- | --- | --- | --- | --- | --- | --- | --- | --- | --- | --- | --- | --- | --- |
| **Resistance Threshold** | 20 | 5 | 5 | 10 | 3 | 50 | 2 | 1.5 | 5 | 50 | 20 | 30 | 100 |
| **10_54** | 16.48 | 1.551 | 3.079 | 2.002 | 3.335 | 63.57 | 0.7305 | 1.032 | 1.832 | 29.22 | 5.844 | 30.61 | 60.14 |
| **36_89** | 16.63 | 4.431 | 4.418 | 8.947 | 3.327 | 79.45 | 5.916 | 1.139 | 1.491 | 21.22 | 3.865 | 30.95 | ND |
| **3D7** | 6.8168 | 2.8094 | 3.6543 | 8.4817 | 2.6474 | 8.8972 | 2.5971 | 0.9846 | 6.6259 | 86.2639 | 20.8618 | 19.0066 | 24.5422 |
| **51** | 29.53 | 3.661 | 3.688 | 9.564 | 4.192 | 108.2 | 3.005 | ND | 2.509 | 20.59 | 7.559 | 41.53 | 125.9 |
| **608** | 15.16 | 3.089 | 3.07 | 4.351 | 6.978 | 93.25 | 2.784 | 1.155 | 1.198 | 10.09 | 5.83 | 10.47 | 104.9 |
| **7G8** | 18.37 | 1.464 | 3.1 | 2.988 | 4.161 | 56.18 | 2.789 | 0.7968 | 1.071 | 31.1 | 6.053 | 40.11 | 38.87 |
| **9_411** | ND | 1.158 | 1.66 | 5.99 | ND | ND | 0.8257 | ND | 1.525 | 5.687 | 0.6979 | 54.52 | ND |
| **CF04.008_1F** | ND | 3.718 | 3.035 | 6.777 | 0.8209 | 6.02 | 2.522 | 1.094 | 3.481 | 36.68 | 4.939 | 18.71 | 82.44 |
| **CF04.009** | ND | 4.1495 | 3.771 | 8.845 | ND | ND | 1.7316 | ND | 9.792 | 72.48 | 10.356 | 29.865 | ND |
| **D10** | 15.2461 | 3.5656 | 7.2633 | 8.7383 | 2.3062 | 11.6735 | 4.4617 | 1.5121 | 10.5989 | 92.63 | 25.4271 | 38.127 | 18.1534 |
| **D6** | 14.92 | 2.076 | 3.138 | 6.612 | 0.6697 | 3.611 | 1.333 | 0.4141 | 6.201 | 29.56 | 6.216 | 3.999 | 4.898 |
| **Dd2** | 10.0602 | 3.4412 | 3.5525 | 9.2125 | 1.2793 | 73.4253 | 2.3138 | 0.8717 | 4.7277 | 74.7528 | 14.9576 | 26.21 | 78.8966 |
| **FCC2** | 9.687 | 3.165 | 3.202 | 8.728 | 1.213 | 9.47 | 4.096 | 1.48 | 7.74 | 156.3 | 35.99 | 24.36 | 27.95 |
| **GA3** | 15.11 | 3.591 | 3.203 | 6.281 | 2.391 | 102.1 | 3.674 | 1.495 | 6.49 | ND | 27.22 | 43.25 | 31.96 |
| **GH2** | 23.3 | 6.718 | 5.983 | 16.78 | 2.366 | 98.03 | 5.694 | 1.707 | 16.03 | 346.7 | 17.82 | 48.54 | 149.1 |
| **HB3** | 8.703 | 1.169 | 3.019 | 3.117 | 1.181 | 9.78 | 0.9405 | 1.413 | 2.764 | 71.51 | 11.18 | 31.22 | 20.28 |
| **IGHCR14** | 4.879 | 0.6559 | 1.416 | 1.202 | 0.4847 | 4.967 | 0.382 | 1.451 | 1.725 | 53.57 | 11.51 | 9.564 | 4.99 |
| **Indochina_I** | 12 | 20.57 | 19.6 | ND | ND | 243.1 | ND | 0.8852 | 4.00E-04 | 6.178 | 3.902 | 28.99 | 153 |
| **JST** | 30.02 | 2.35 | 5.658 | 3.838 | 2.193 | 129.1 | 1.698 | 1.203 | 1.296 | 22.07 | 6.811 | 41.34 | 24.52 |
| **K1** | 16.07 | 1.902 | 3.124 | 3.176 | 2.442 | 86.42 | 3.509 | 1.52 | 1.907 | 30.84 | 13.57 | 35.78 | 77.81 |
| **M24** | 7.63 | 4.6 | 4.43 | 5.546 | 1.524 | 13.09 | 2.49 | 1.158 | 1.919 | 59.81 | 13.48 | 14.23 | 55.33 |
| **Malayan_Camp** | 7.944 | 2.887 | 1.811 | 1.775 | 1.171 | 7.279 | 2.319 | 1.126 | 1.283 | ND | 6.983 | 31.26 | 8.196 |
| **Muz51.1** | 19.64 | 3.29 | 3.18 | 7.449 | 1.939 | 60.44 | 2.271 | 1.13 | 1.696 | 19.5 | 8.501 | 28.23 | 39.73 |
| **PR145** | 11.11 | 15.79 | 13.66 | 29.7 | 6.59 | 51.24 | 12.02 | 1.106 | 16 | 140.8 | 53.43 | 31.58 | 149.1 |
| **RAJ116** | 19.23 | 1.075 | 1.805 | 1.367 | 1.315 | 68.59 | 1.035 | 1.546 | 0.0049 | 6.418 | 4.105 | 34.31 | 4.36 |
| **RO33** | 11.3737 | 2.6614 | 6.0825 | 6.9751 | 1.8615 | 11.0702 | 2.8762 | 1.6415 | 3.1416 | 69.66 | 8.094 | 34.0955 | 13.4901 |
| **Santa_Lucia** | 20.44 | 3.629 | 4.61 | 8.644 | 0.3903 | 11.76 | 3.567 | 1.171 | 0.7153 | 30.34 | 5.822 | 38.02 | 260.1 |
| **SenP05.02** | 16.24 | 5.695 | 3.178 | 11.25 | 2.493 | ND | 3.518 | 1.52 | 0.9853 | 30.43 | 6.798 | 29 | ND |
| **SenP08.04** | 17.33 | 4.873 | 4.15 | 11.12 | 1.19 | 14.85 | 3.015 | 0.4386 | 11.72 | 62.21 | 26.03 | 7.81 | 44.76 |
| **SenP09.04** | 4.517 | 5.332 | 4.177 | 15.7 | 0.7054 | 8.312 | 5.092 | 1.404 | 9.234 | 174 | 25.44 | 14.78 | 54.86 |
| **SenP11.02** | 92.05 | 12.75 | 11.15 | 19.99 | 1.881 | 25.83 | 8.258 | 0.7377 | 17.35 | 88.75 | 44.76 | 14.44 | 94.63 |
| **SenP19.04** | 7.157 | 11.66 | 10.98 | 24.04 | ND | 11.4 | 9.225 | 1.858 | 14.71 | 95.17 | 50.83 | 30.21 | ND |
| **SenP26.04** | 44.23 | 12.71 | 3.23 | 14.82 | 1.143 | 40.38 | 8.889 | 0.4341 | 20.6 | 57.79 | 84.86 | 5.288 | ND |
| **SenP27.02** | 9.269 | 3.232 | 3.635 | 8.222 | ND | 9.813 | 2.664 | 1.258 | 1.83 | 31.2 | 5.972 | 21.2 | ND |
| **SenP31.01** | 6.961 | 1.991 | 1.999 | 6.131 | 0.4997 | 8.854 | 2.966 | 1.214 | 3.177 | 78.89 | 17.03 | 22.56 | ND |
| **SenP51.02** | 29.62 | 5.041 | 4.748 | 8.875 | 0.3225 | 62.05 | 3.398 | 1.627 | 3.319 | 48.55 | 8.601 | 34.89 | 50.08 |
| **SenP60.02** | 15.8 | 4.084 | 4.978 | 9.62 | 0.9355 | 99.93 | 3.854 | 1.255 | 1.616 | 21.99 | 11.93 | 21.19 | 47.78 |
| **SenT15.04** | ND | 3.595 | 3.016 | 6.9315 | ND | ND | 3.3665 | ND | 5.262 | 51.32 | 3.948 | 49.53 | ND |
| **SenT26.04** | 16.05 | 3.149 | 3.116 | 4.827 | 1.19 | 79.98 | 2.991 | 1.43 | 2.905 | 30.92 | 7.877 | 24.72 | 121.1 |
| **SenT28.04** | 11.9 | 3.744 | 3.139 | 7.846 | 1.1 | 51.01 | 2.343 | 1.461 | 7.5 | 62.69 | 32.64 | 15.63 | 139.6 |
| **SenV34.04** | 21.39 | 5.1 | 4.645 | 6.781 | 0.5187 | ND | 3.024 | 1.627 | 2.37 | 30.34 | 4.018 | 28.48 | ND |
| **SenV35.04** | 5.567 | 3.078 | 3.68 | 4.706 | 0.8165 | 9.364 | 5.374 | 1.359 | 2.761 | 45.52 | 20.6 | 26.28 | ND |
| **SenV42.05** | 6.456 | 1.06 | 1.565 | 1.717 | 0.7363 | 9.594 | 1.731 | 1.016 | 4.476 | 13.6 | 10.04 | 4.485 | ND |
| **TD203** | 15.63 | 8.15 | 4.736 | 20.56 | 8.49 | 52.86 | 8.507 | 1.071 | 7.938 | 156.2 | 30.32 | 39.2 | 67.53 |
| **TD257** | 11.68 | 11.88 | 8.532 | 24.53 | 2.215 | 55.41 | 11.63 | 1.539 | 15.96 | 227.6 | 50.42 | 35.53 | 253.5 |
| **TM327** | 9.891 | 6.032 | 1.7 | 8.862 | 1.146 | 41.12 | 3.851 | 2.022 | 15.49 | 249.3 | 41.25 | 50.89 | 70.72 |
| **TM90C2A** | 18.85 | 16.17 | 10.31 | 28.32 | 3.704 | 58.94 | 5.376 | 1.086 | 12.51 | 264.5 | 28.04 | 30.04 | 209.1 |
| **TM91C235** | ND | 12.43 | 12.46 | 17.66 | ND | ND | 7.409 | ND | 70.65 | 154.5 | 57.96 | 12.69 | ND |
| **V1/S** | 17.98 | 1.625 | 3.459 | 6.172 | 4.02 | 155.5 | 1.035 | 1.538 | 1.739 | 26.4 | 12.84 | 38.58 | 224.3 |

# 
