# Supplementary material for: Identification and Functional Validation of the Novel Antimalarial Resistance Locus PF10_0355 in Plasmodium falciparum
Source: PLoS Genet. 2011 Apr 21;7(4):e1001383. doi: 10.1371/journal.pgen.1001383 (PMC3080868; doi:10.1371/journal.pgen.1001383)
Supplement: Table S5 — Parasites used in the GWAS. Parasites used, indicating their nucleotide and amino acid sequence for various positions (indicated by number) in the dhfr, pfcrt, and pfmdr1 gene loci. (0.24 MB DOC) [file pgen.1001383.s020.doc]

|  | ***dhfr*** | | | | | | | | | | | | | ***pfcrt*** | | | | | | | | | | | | ***pfmdr1*** | | | | | | | | | | |
| --- | --- | --- | --- | --- | --- | --- | --- | --- | --- | --- | --- | --- | --- | --- | --- | --- | --- | --- | --- | --- | --- | --- | --- | --- | --- | --- | --- | --- | --- | --- | --- | --- | --- | --- | --- | --- |
|  | *Nucleotide Sequence at AA Position* | | | | | | *Amino Acid Sequence* | | | | | | | *Nucleotide Sequence at AA Position* | | | | | *Amino Acid Sequence* | | | | | | | *Nucleotide Sequence at AA Position* | | | | | | *Amino Acid Sequence* | | | | |
| *Parasite* | 16 | 51 | 59 | 108 | 164 | 506 | | 16 | 51 | 59 | 108 | 164 | 506 | | 72-76 | 326 | 356 | 371 | 72 | 74 | 75 | 76 | 326 | 356 | 371 | | 86 | 184 | 1034 | 1042 | 1246 | 86 | 184 | 1034 | 1042 | 1246 |
| **51** | C | T | T | A | A | T | | A | I | C | N | I | Y | | TCTGTAATGAATACA | aac | Tta | aGa | S | M | N | T | N | L | R | | A | T | Tgt | Gat | Tat | N | F | C | D | Y |
| **10_54** | C | T | T | A | A | T | | A | I | C | N | I | Y | | TCTGTAATGAATACA | aac | Tta | aGa | S | M | N | T | N | L | R | | A | T | Tgt | Gat | Tat | N | F | C | D | Y |
| **36_89** | C | T | T | A | A | T | | A | I | C | N | I | Y | | TCTGTAATGAATACA | aac | Tta | aGa | S | M | N | T | N | L | R | | A | T | Tgt | Gat | Tat | N | F | C | D | Y |
| **3D7** | C | A | T | G | A | T | | A | N | C | S | I | Y | | TGTGTAATGAATAAA | aac | ata | aga | C | M | N | K | N | I | R | | A | A | agt | aat | gat | N | Y | S | N | D |
| **7G8** | C | T | T | A | A | T | | A | I | C | N | I | Y | | AGTGTAATGAATACA | aac | Tta | aGa | S | M | N | T | N | L | R | | A | T | Tgt | Gat | Tat | N | F | C | D | Y |
| **9_411** | C | T | T | G | A | T | | A | I | C | N | I | Y | | TCTGTAATGAATACA | aac | Tta | aGa | S | M | N | T | N | L | R | | A | T | Tgt | Gat | Tat | N | F | C | D | Y |
| **A4** | C | T | C | A | A | T | | A | I | C | T | I | Y | | TGTGTAATTGAAACA | aac | Ata | aTa | C | I | E | T | N | I | I | | T | A | Agt | Aat | Gat | Y | Y | S | N | D |
| **APO41** | C | T | C | A | A | T | | A | I | R | N | I | Y | | TGTGTAATTGAAACA | aac | Ata | aTa | C | I | E | T | N | I | I | | A | T | Agt | Aat | Gat | N | F | S | N | D |
| **CF04.008_12G** | C | T | T | A | A | T | | A | N | R | N | I | Y | | TGTGTAATTGAAACA | aac | Ata | aTa | C | M | N | A | N | I | I | | T | A | Agt | Aat | Gat | Y | Y | S | N | D |
| **CF04.008_1F** | C | T | C | A | A | T | | A | I | R | N | I | Y | | TGTGTAATGAATAAA | aac | Ata | aGa | C | M | N | A | N | I | R | | T | A | Agt | Aat | Gat | Y | Y | S | N | D |
| **CF04.009** | C | T | C | A | A | T | | A | I | R | N | I | Y | | TGTGTAATGAATAAA | aac | Ata | aGa | C | M | N | A | N | I | R | | A | T | Agt | Aat | Gat | N | F | S | N | D |
| **D10** | C | A | T | G | A | T | | A | N | C | S | I | Y | | TGTGTAATGAATAAA | aac | Ata | aGa | C | M | N | K | N | I | R | | A | A | Agt | Aat | Gat | N | Y | S | N | D |
| **D6** | C | A | T | G | A | T | | A | N | C | S | I | Y | | TGTGTAATGAATAAA | aac | Ata | aGa | C | M | N | K | N | I | R | | A | A | Agt | Aat | Gat | N | Y | S | N | D |
| **Dd2** | C | T | C | A | A | T | | A | I | R | N | I | Y | | TGTGTAATTGAAACA | aac | Ata | aTa | C | I | E | T | N | T | I | | T | A | Agt | Aat | Gat | Y | Y | S | N | D |
| **FCC2** | C | A | T | A | A | T | | A | N | C | N | I | Y | | TGTGTAATGAATAAA | aac | Ata | aGa | C | M | N | K | N | I | R | | A | A | Agt | Aat | Gat | N | Y | S | N | D |
| **GH2** | C | T | C | A | A | T | | A | I | R | N | I | Y | | TGTGTAATTGAAACA | aac | Ata | aTa | C | I | E | T | N | I | I | | A | A | Agt | Aat | Gat | N | Y | S | N | D |
| **HB3** | C | A | T | G | A | T | | A | N | C | S | I | Y | | TGTGTAATGAATAAA | aac | Ata | aGa | C | M | N | K | N | I | R | | A | T | Agt | Gat | Gat | N | F | S | D | D |
| **Indochina_I** | C | T | C | A | A | T | | A | I | R | N | I | Y | | TGTGTAATTGAAACA | aac | Ata | aTa | C | I | E | T | N | I | I | | A | T | Tgt | Gat | Tat | N | F | C | D | Y |
| **JST** | C | T | T | A | A | T | | A | N | C | N | I | Y | | TCTGTAATGAATACA | aac | Tta | aGa | S | M | N | T | N | L | R | | A | A | Tgt | Gat | Tat | N | Y | C | D | Y |
| **K1** | C | A | C | A | A | T | | A | N | R | N | I | Y | | TGTGTAATTGAAACA | aac | Ata | aTa | C | I | E | T | N | I | I | | T | A | Agt | Aat | Gat | Y | Y | S | N | D |
| **M24** | C | A | T | G | A | T | | A | N | C | S | I | Y | | TGTGTAATGAATAAA | aac | Ata | aGa | C | M | N | K | N | I | R | | A | T | Agt | Aat | Gat | N | F | S | N | D |
| **Malayan Camp** | C | A | T | A | A | T | | A | N | C | N | I | Y | | TGTGTAATGAATAAA | aac | Ata | aGa | C | M | N | K | N | I | R | | A | T | Agt | Aat | Gat | N | Y | S | N | D |
| **Muz51.1** | C | A | T | G | A | T | | A | N | C | S | I | Y | | AGTGTAATGAATACA | aac | Tta | aGa | S | M | N | T | N | L | R | | T | A | Agt | Aat | Gat | Y | Y | S | N | D |
| **PR145** | C | T | C | A | T | T | | A | I | R | N | I | Y | | GGTGTAATTGAAACA | aac | Ata | aTa | G | I | E | T | N | I | I | | A | T | Agt | Aat | Gat | N | F | S | N | D |
| **PS189** | C | A | T | G | A | T | | A | N | C | S | I | Y | | TGTGTAATGAATAAA | aac | Ata | aGa | C | M | N | K | N | I | R | | A | T | Agt | Aat | Gat | N | F | S | N | D |
| **RO33** | C | A | C | A | A | T | | A | N | R | N | I | Y | | TGTGTAATGAATAAA | aac | Ata | aGa | C | M | N | K | N | I | R | | T | T | Agt | Aat | Gat | Y | F | S | N | D |
| **Santa Lucia** | G | A | T | G | A | T | | A | N | C | S | I | Y | | TGTGTAATGAATAAA | aac | Ata | aGa | C | M | N | T | N | I | R | | A | T | Tgt | Gat | Gat | N | F | C | D | D |
| **SenP05.02** | C | T | C | A | A | T | | A | N | C | N | I | Y | | TGTGTAATTGAAACA | aac | Ata | aTa | C | I | E | T | N | I | I | | T | T | Agt | Aat | Gat | Y | F | S | N | D |
| **SenP08.04** | C | T | C | A | A | T | | A | I | R | N | I | Y | | TGTGTAATGAATAAA | aac | Ata | aGa | C | M | N | K | N | I | R | | A | T | Agt | Aat | Gat | N | F | S | N | D |
| **SenP09.04** | C | T | C | A | A | T | | A | I | R | N | I | Y | | TGTGTAATGAATAAA | aac | Ata | aGa | C | M | N | K | N | I | R | | A | A | Agt | Aat | Gat | N | Y | S | N | D |
| **SenP11.02** | C | A | T | G | A | T | | A | N | C | S | I | Y | | TGTGTAATGAATAAA | aac | Ata | aGa | C | M | N | K | N | I | R | | A | T | Agt | Aat | Gat | N | F | S | N | D |
| **SenP19.04** | C | T | C | A | A | T | | A | N | R | N | I | Y | | TGTGTAATGAATAAA | aac | Ata | aGa | C | M | N | K | N | I | R | | A | A | Agt | Aat | Gat | N | Y | S | N | D |
| **SenP26.04** | C | T | C | A | A | T | | A | N | R | N | I | Y | | TGTGTAATTGAAACA | aac | Ata | aTa | C | I | E | T | N | I | I | | T | T | Agt | Aat | Gat | Y | F | S | N | D |
| **SenP27.02** | C | T | C | A | A | T | | A | I | R | N | I | Y | | TGTGTAATGAATAAA | aac | Ata | aGa | C | M | N | K | N | I | R | | T | T | Agt | Aat | Gat | Y | F | S | N | D |
| **SenP31.01** | C | A | T | G | A | C | | A | N | C | S | I | Y | | TGTGTAATGAATAAA | aac | Ata | aGa | C | M | N | K | N | I | R | | A | T | Agt | Aat | Gat | N | F | S | N | D |
| **SenP51.02** | C | T | C | A | A | T | | A | I | R | N | I | Y | | TGTGTAATTGAAACA | aac | Ata | aTa | C | I | E | T | N | I | I | | T | T | Agt | Aat | Gat | Y | F | S | N | D |
| **SenP60.02** | C | A | T | G | A | T | | A | N | C | S | I | Y | | TGTGTAATGAATAAA | aac | Ata | aGa | C | M | N | K | N | I | R | | A | T | Agt | Aat | Gat | N | F | S | N | D |
| **SenT15.04** | C | T | C | A | A | T | | A | I | R | N | I | Y | | TGTGTAATTGAAACA | aac | Ata | aTa | C | I | E | T | N | I | I | | A | T | Agt | Aat | Gat | N | F | S | N | D |
| **SenT26.04** | C | T | C | A | A | T | | A | T | R | N | I | Y | | TGTGTAATTGAAACA | aac | Ata | aTa | C | I | E | T | N | I | I | | T | T | Agt | Aat | Gat | Y | F | S | N | D |
| **SenT28.04** | C | T | C | A | A | T | | A |  | R | N | I | Y | | TGTGTAATTGAAACA | aac | Ata | aTa | C | I | E | T | N | I | I | | A | T | Agt | Aat | Gat | N | F | S | N | D |
| **SenV34.04** | C | A | T | G | A | T | | A | N | C | S | I | Y | | TGTGTAATTGAAACA | aac | Ata | aTa | C | I | E | T | N | I | I | | T | T | Agt | Aat | Gat | Y | F | S | N | D |
| **SenV35.04** | C | A | T | G | A | T | | A | N | C | S | I | Y | | TGTGTAATGAATAAA | aac | Ata | aGa | C | M | N | K | N | I | R | | A | T | Agt | Aat | Gat | N | F | S | N | D |
| **SenV42.05** | C | T | C | A | A | T | | A | I | C | N | I | Y | | TGTGTAATTGAAACA | aac | Ata | aTa | C | I | E | T | N | I | I | | A | A | Agt | Aat | Gat | N | Y | S | N | D |
| **T2_C6** | C | A | T | G | A | T | | A | N | R | S | I | Y | | TGTGTAATGAATAAA | aac | Ata | aGa | C | M | N | K | N | I | R | | A | A | Agt | Aat | Gat | N | Y | S | N | D |
| **TD203** | C | T | C | A | T | T | | A | I | C | N | I | Y | | TGTGTAATTGAAACA | aac | Ata | aTa | C | I | E | T | N | I | I | | A | T | Agt | Aat | Gat | N | F | S | N | D |
| **TD257** | C | T | C | A | T | T | | A | I | C | N | I | Y | | TGTGTAATTGAAACA | aac | Ata | aTa | C | I | E | T | N | I | I | | A | T | Agt | Aat | Gat | N | F | S | N | D |
| **TM327** | C | T | C | A | T | T | | A | I | C | N | I | Y | | TGTGTAATTGAAACA | aac | Ata | aTa | C | I | E | T | N | I | I | | A | T | Agt | Aat | Gat | N | F | S | N | D |
| **TM345** | C | T | C | A | T | T | | A | I | C | N | I | Y | | TGTGTAATTGAAACA | aac | Ata | aTa | C | I | E | T | N | I | I | | A | T | Agt | Aat | Gat | N | F | S | N | D |
| **TM90C2A** | C | T | C | A | T | T | | A | I | C | N | I | Y | | TGTGTAATTGAAACA | aac | Ata | aTa | C | I | E | T | N | I | I | | A | T | Agt | Aat | Gat | N | F | S | N | D |
| **TM90C6A** | C | T | C | A | T | T | | A | I | C | N | I | Y | | TGTGTAATTGAAACA | aac | Ata | aTa | C | I | E | T | N | I | I | | A | A | Agt | Aat | Gat | N | Y | S | N | D |
| **TM91C235** | C | T | C | A | T | T | | A | I | C | N | I | Y | | TGTGTAATTGAAACA | aac | Ata | aTa | C | I | E | T | N | I | I | | A | T | Agt | Aat | Gat | N | F | S | N | D |
| **V1/S** | C | T | C | A | T | T | | A | I | C | N | I | Y | | TGTGTAATTGAAACA | aac | Ata | aTa | C | I | E | T | N | I | I | | T | A | Agt | Aat | Gat | Y | Y | S | N | D |

# 
