# Supplementary material for: Identification and Functional Validation of the Novel Antimalarial Resistance Locus PF10_0355 in Plasmodium falciparum
Source: PLoS Genet. 2011 Apr 21;7(4):e1001383. doi: 10.1371/journal.pgen.1001383 (PMC3080868; doi:10.1371/journal.pgen.1001383)
Supplement: Table S6 — PF10_0355 copy number summary for 38 parasites tested by qPCR using the Delta Delta Ct method. Copy number (CN) was compared to the reference locus PF07_0076 and 3D7 was used as a reference strain. A cut-off of 1.4 was used to define PF10_0355 copy number greater than 1; parasites with greater than 1 copy of PF10_0355 are shaded. Parasites are ranked by Halofantrine (HFN) IC50: HFN-sensitive parasites are indicated by an S and HFN-resistant parasites are indicated by an R. (0.08 MB DOC) [file pgen.1001383.s021.doc]

| **Parasite** | **CN** | **HFN** |
| --- | --- | --- |
| Indochina_I | 0.92 | S |
| RAJ116 | 0.92 | S |
| Santa_Lucia | 1.03 | S |
| SenP05.02 | 0.98 | S |
| 7G8 | 0.74 | S |
| Malayan_Camp | 0.94 | S |
| JST | 1.06 | S |
| 36_89 | 1.07 | S |
| SenP60.02 | 1.19 | S |
| Muz51.1 | 0.64 | S |
| IGHCR14 | 1.63 | S |
| V1/S | 0.89 | S |
| 10_54 | 0.99 | S |
| K1 | 0.87 | S |
| M24 | 0.76 | S |
| SenV34.04 | 4.92 | S |
| 51 | 0.77 | S |
| SenV35.04 | 0.86 | S |
| HB3 | 0.88 | S |
| RO33 | 1.26 | S |
| SenP31.01 | 0.77 | S |
| SenP51.02 | 1.09 | S |
| CF04.008_1F | 0.95 | S |
| SenV42.05 | 1.26 | S |
| Dd2 | 1.07 | S |
| SenT15.04 | 1.71 | R |
| 3D7 | 1 | R |
| SenT28.04 | 0.73 | R |
| FCC2 | 0.76 | R |
| TD203 | 0.9 | R |
| D10 | 1.06 | R |
| SenP08.04 | 0.95 | R |
| TM90C2A | 1.43 | R |
| SenP19.04 | 1.06 | R |
| PR145 | 0.94 | R |
| GH2 | 1.71 | R |
| SenP11.02 | 7.14 | R |
| SenP26.04 | 1.68 | R |
