# Supplementary material for: Identification and Functional Validation of the Novel Antimalarial Resistance Locus PF10_0355 in Plasmodium falciparum
Source: PLoS Genet. 2011 Apr 21;7(4):e1001383. doi: 10.1371/journal.pgen.1001383 (PMC3080868; doi:10.1371/journal.pgen.1001383)
Supplement: Table S7 — Annotation and GeneID Information for identified genes in Figure 1B. (0.05 MB DOC) [file pgen.1001383.s022.doc]

| **GeneID** | **π** | **F_ST_** | **Annotation** | **Category** | **Tag** |
| --- | --- | --- | --- | --- | --- |
| **MAL8P1_23** | 1.54E-04 | 0.646 | ubiquitin-protein ligase 1, putative | enzymes, ACS and transporters | UBQ Ligase |
| **PF13_0201** | 6.39E-03 | 0.216 | thrombospondin-related anonymous protein, TRAP | other | TRAP |
| **PFA0650w** | 4.37E-03 | 0.323 | surface-associated interspersed gene pseudogene, (SURFIN) pseudogene | antigens, var, rifin, stevor, surfin | SURFIN |
| **PF08_0105** | 6.10E-03 | 0.204 | rifin | antigens, var, rifin, stevor, surfin | Rifin |
| **PFB0960c** | 4.31E-03 | 0.036 | P. falciparum Maurer’s Cleft 2 transmembrane domain protein 2.1, PfMC-2TM_2.1 | other | Mauer’s Cleft |
| **MAL7P1_27** | 6.36E-04 | 0.387 | chloroquine resistance transporter | enzymes, ACS and transporters | PFCRT |
| **PF10_0345** | 6.52E-03 | 0.240 | merozoite surface protein 3 | antigens, var, rifin, stevor, surfin | MSP3 |
| **PFI1475w** | 1.95E-03 | 0.221 | merozoite surface protein 1, precursor | antigens, var, rifin, stevor, surfin | MSP1 |
| **PFB0972w** | 9.90E-03 | 0.077 | hypothetical protein | other | * |
| **PFL0030c** | 7.95E-03 | 0.050 | erythrocyte membrane protein 1 (PfEMP1) | antigens, var, rifin, stevor, surfin | Var2CSA |
| **PFD0830w** | 5.96E-04 | 0.459 | bifunctional dihydrofolate reductase-thymidylate synthase | enzymes, ACS and transporters | DHFR |
| **PF11_0344** | 6.46E-03 | 0.074 | apical membrane antigen 1, AMA1 | antigens, var, rifin, stevor, surfin | AMA1 |
| **PF10_0051** | 5.32E-03 | 0.215 | ADP/ATP carrier protein, putative | enzymes, ACS and transporters | ADP/ATP Carrier |
| **PFB0685c** | 5.75E-04 | 0.497 | acyl-CoA synthetase, PfACS9 | enzymes, ACS and transporters | ACS9 |
| **PFF1350c** | 2.00E-03 | 0.584 | acetyl-coenzyme a synthetase | enzymes, ACS and transporters | ACS |
| **PFE1250w** | 1.66E-03 | 0.602 | acyl-CoA synthetase, PfACS10 | enzymes, ACS and transporters | ACS10 |
