# Supplementary material for: Identification and Functional Validation of the Novel Antimalarial Resistance Locus PF10_0355 in Plasmodium falciparum
Source: PLoS Genet. 2011 Apr 21;7(4):e1001383. doi: 10.1371/journal.pgen.1001383 (PMC3080868; doi:10.1371/journal.pgen.1001383)
Supplement: Text S1 — Supplemental methods. (1.29 MB PDF) [file pgen.1001383.s023.pdf]

Supplemental Materials for Identification and Functional Validation of the Novel Antimalarial Resistance Locus *PF10_0355* in *Plasmodium falciparum*, Van Tyne, et al.

Supplemental Methods

Parasites

Parasites (Table S1) were obtained from the Malaria Research and Reagent Resource Repository (MR4, malaria.mr4.org) or additional sources noted (Supplemental Methods, Table S1, Acknowledgements). The following parasite lines were obtained through MR4: parasite line 3D7 (MRA-151, deposited by D. Walliker); parasite line 7G8 (MRA-152, deposited by D. Walliker); parasite line HB3 (MRA-155, deposited by T.E. Wellems); parasite line Dd2 (MRA-156, deposited by T.E. Wellems); parasite line K1 (MRA-159, deposited by D.E. Kyle); parasite line V1/S (MRA-176, deposited by D.E. Kyle); parasite line RO-33 (MRA-200, deposited by D. Walliker, U. Certa and R. Reber-Liske); parasite line D10 (MRA-201, deposited by Y. Wu); parasite line TM90C2A (MRA-202, deposited by D.E. Kyle); parasite line TM90C6A (MRA-205, deposited by D.E. Kyle); parasite line TM91C235 (MRA-206, deposited by D.E. Kyle); parasite line WR87 (MRA-284, deposited by D.E. Kyle); parasite line D6 (MRA-285, deposited by D.E. Kyle); parasite line Malayan Camp (MRA-330, deposited by L.H. Miller and D. Baruch); parasite line Indochina I (MRA-347, deposited by W.E. Collins); parasite line Santa Lucia (MRA-362, deposited by W.E. Collins); parasite line FCC-2 (MRA-733, deposited by W. Trager); and parasite line T2-C6 (MRA-818, deposited by X. Su). Patient samples were obtained as part of ongoing studies in Senegal and Malawi described elsewhere in accordance with human subject guidelines. Additional parasites were the kind gift of: Alejandro Miguel Katzin (51, 10_54, 36_89, and 9_411); Christian Happi (APO41); Abdoulaye Djimde and Chris Plowe (PS189); Joseph Smith (A4); Karen Day (Muz51.1); Dennis Kyle and Sodsri Thaithong (TD203, TD257, TM327, TM345, GH2, and PR145); Sandra do Lago Moraes (JST); and Xin-zhuan Su (MR24). DNA from *P. reichenowi* was kindly provided by John Barnwell.

PCR Genotyping

Genomic regions (458850-459204) surrounding the *pfcrt* (MAL7P1.27) locus was amplified using the polymerase chain reaction using oligonucleotide primer sequences (CCTTGTCGACCTTAACAGATG, CTATTCCACCTACCAATATAAAAC) and the resulting DNA sample was sequenced using standard methods. In a similar manner the genomic region (754984-755584) surrounding the *dhfr* locus (PFD0830w) (oligonucleotide primer sequences: CAAGATTGATACATAAAGATAATAT, TTCTTGATAAACAACGGAACCTCCT); and the *pfmdr1* locus (PFE1150w) (oligonucleotide primer sequences: TGTTGAAAGATGGGTAAAGAGCAGAAAGAG, TACTTTCTTATTACATATGACACCACAAACA) were utilized [1].

SNP Discovery

The SNP discovery methodology was similar to those described in Volkman 2007 [2]. 1X ABI shotgun sequence was obtained for nine geographically diverse parasite isolates that were previously sequenced to 0.25X coverage, bringing total coverage to 1.25X per isolate. These nine isolates include: 7G8, Santa Lucia (El Salvador), V1/S, D10, FCC-2/Hainan, D6, RO-33, Senegal V34.04 and K1. Three of the twelve previously sequenced isolates in Volkman 2007 were excluded from additional sequencing, as they were previously found to be nearly genetically identical, suggesting possible contamination in culture [2]. Reads ends with low quality (PHRED <10) bases were trimmed. Reads less than 100 bases, containing greater than 3% internal N's, or containing a mononucleotide repeat covering greater than 80% of the read were discarded. Reads were aligned to the PlasmoDB version 5 of the 3D7 genome using BLAT37 [3] requiring 95% identity, a minimum score of 100, less than 20% gaps, and coverage of at least half of the read. Only the highest scoring alignment for each read was kept and paired reads which aligned more than 10kb apart or in the wrong orientation were discarded. The Neighborhood Quality Standard (NQS) algorithm was used to distinguish real polymorphisms from sequence errors [4]. We required the SNP to have a minimum quality score of 25, and the five base neighborhood to have a minimum score of 20. We allowed one mismatch and no indels in the neighborhood. We discarded SNPs when another read from the same sample met the NQS criteria at that position but did not have a sequence difference.

Array Development and Assessment

Based on all 111,536 discovered SNPs [2,5,6] in *P. falciparum*, and given design parameters and unique sequence constraints, we were able to design assays for 74,656 markers. Each of 74,656 SNPs is represented by a probe set of 12 to 84 probes, for a total of 4.4 million genotyping probes on the Affymetrix 49-format array. These were hybridized to 63 unique samples (totaling 81 arrays with replicates). Genotype calls were produced using the BRLMM-P algorithm, a variant of the RLMM algorithm [7], included in Affymetrix Power Tools version 1.8.5, and clustered over all 81 arrays. BRLMM-P was forced into a haploid calling mode by setting assigning all SNPs to the “Y chromosome” and setting all arrays to “male”.

The array with sample TM93C1088 is eliminated immediately after clustering (arbitrarily, since the chip claiming to be TM90C6A and the chip claiming to be TM93C1088 are identical). We also remove samples CF04.010 and Senegal Th10.04, which were suspected to be multi-clonal based upon molecular barcode analysis [8]. A halofuginone-resistant version of Dd2, a human-DNA sample, and the *P. reichenowi* ancestral samples are also removed at this stage, leaving 57 unique samples (totaling 75 arrays with replicates) for analysis. We then calculate a call rate for each SNP and remove 7,778 SNPs that have below an 80% call rate, leaving 66,878 SNPs. Since technical replicates showed 99.9% repeatability between chips, we merged replicate data for each of the 57 samples, producing a no-call when the replicates indicated discordant genotypes.

Concordance against sequencing data was calculated in both major and minor alleles for 17 sequenced reference strains [9]. The following 17 samples were compared against sequencing data for concordance: 3D7, Dd2, FCC-2, Malayan Camp, D10, K1, V1/S, RO33, D6, Senegal P31.01, Senegal P51.02, Senegal V34.04, Senegal V35.04, 7G8, A4 (subclone of IT04 [10]), Santa Lucia, and HB3. These are the 18 parasites presented in Fig 1 of Volkman 2007 [2], removing the three found to be

genetically identical, and adding the two strains 3D7 and A4. A total of 18,303 SNPs lacked call overlap between array genotypes and sequencing genotypes in minor alleles and were thus removed, since concordance in both alleles could not be fully calculated. Another 30,993 SNPs were removed due to imperfect concordance, and of these discordant SNPs, most (28,789) exhibited monomorphic behavior on the array, suggesting that much of the discordance may be attributed to either a faulty assay or false discovery. The remaining 17,582 perfectly concordant SNPs constituted the high confidence set of assays used in our analyses.

Copy Number Variation (CNV) Analysis

We examined the ability to detect copy number variants (CNV) using the array by first studying a known CNV using the hybridization intensity signal of the SNP genotyping probes on the array. Kidgell, et al. (2006) [11] reported that the *pfmdr1* locus was present in 3-4 copies in the Dd2 strain relative to a collection of other strains. We compared *Z*-scores of the normalized hybridization intensity of perfect match probes for SNPs in the neighborhood of *pfmdr1* for Dd2 and six parasites estimated by Kidgell, et al. to contain only 1 copy of the locus (3D7, 7G8, HB3, D10, D6, K1). For each SNP assay we utilized the average hybridization intensity of all perfect-match probes. Hybridization intensity values were background corrected and normalized to reduce inter-array variation artifacts. SNPs with a hybridization intensity standard deviation equal to or greater than half the magnitude of the average hybridization intensity across all arrays were excluded from analysis. Figure S11 illustrates that probes for many of the SNPs assayed within the *pfmdr1* locus exhibit notably higher hybridization intensity values in Dd2 relative to the other parasites, with 13 assays exhibiting average intensities greater than 2 standard deviations higher than observed in the other parasites

Genome-wide Association Study (GWAS)

We performed GWAS for drug resistance to thirteen antimalarials: amodiaquine (ADQ), artemether (ARTM), artesunate (ARTN), artemisinin (ARTS), atovaquone (ATV), chloroquine (CQ), dihydroartemisinin (DHA), halofuginone (HFG), halofantrine (HFN), lumefantrine (LUM), mefloquine (MFQ), piperaquine (PIP) and quinine (QN). 50 out of 59 samples had drug phenotype data. IC_{50} data are shown in Table S4 and Figure S13 for these 50 parasites against the 13 drugs.

The following drugs were obtained from Sigma Aldrich: artemisinin, dihydroartemisinin, chloroquine, mefloquine, and quinine. The following were obtained from AK Scientific: artemether, artesunate, halofantrine, lumefantrine, and piperaquine. The following were obtained from USP: amodiaquine and atovaquone. Each drug was tested in triplicate for each parasite. Additionally, some parasites were tested with multiple biological replicates: 3D7 (nine biological replicates per drug, each in triplicate), Dd2 (three replicates) and RO-33, D10, and 207-89 (two replicates).

SNPs were filtered down to a set that contained at least 5 strains with a minor allele as well as an 80% call rate under every phenotype condition. The final data set includes 7,437 SNPs. This gives us a genome-wide significance threshold of $-\log_{10}(P\text{-value}) > 5.17$ by Bonferroni correction for multiple testing. For binary phenotype tests (Fisher's exact test, Fisher's permuted, CMH, and HLR), we used IC_{50} cutoffs shown in Table S4. For tests requiring defined geographic clusters (CMH, HLR, Fisher's permuted), the three population clusters are defined by PCA, as in the LRH analysis, and the assignments are shown in Table S1.

Pointwise *P*-values were computed using PLINK [12]. Quantile-quantile plots (qq-plots) were used to examine the resulting *P*-value distributions for inflating effects due to population structure (Fig. S7). Because most of the genome is assumed to fit the null hypothesis (most of the genome should not be in association with the phenotype), significant, early deviations from this expectation may result in a high false positive rate. The null expectation is plotted as the unity diagonal line in Figure S7. Bonferroni significance is plotted as the dashed line and Benjamini-Hochberg significance is marked with the dotted line. Since most Fisher's results show evidence of inflation, we do not report these results in Figure 2 or Table 1.

Permutations of Fisher's exact test can be used to compute empirical pointwise *P*-values based on a simulated null distribution. We used PLINK to perform this permutation while respecting the phenotype frequencies present in our three predefined population clusters. The resulting *P*-value distributions (Fig. S7) do not show inflation due to population structure, however no significant hits were found for any drug.

Similarly, the Cochran-Mantel-Haenszel (CMH) test can perform population-stratified analyses for association. We used PLINK to compute *P*-values (Fig. S7), and again, we see appropriate corrections for population structure, but no hits reach genome-wide (Bonferroni) significance.

The Efficient Mixed-Model Association (EMMA) test was specifically designed to handle quantitative trait associations to a data set with complex population structure using a linear mixed model [13]. It calculates a genotype similarity matrix instead of discrete categories and does not require *a priori* specification of population structure. The resulting *P*-value distributions demonstrate little remaining effect from population structure (Fig. S8) while retaining power to find a number of associations at genome-wide significance (Fig. S8, 2A, Table 1).

The Haplotype Likelihood Ratio (HLR) test is a multi-marker association test [14]. Unlike a standard, χ^2-based multi-marker test which looks for differences in haplotype frequencies in cases vs. controls, the HLR test specifically models the likelihood that a single haplotype rose to dominance in cases while all other haplotypes proportionally decreased. It produces a LOD score, which is the maximum likelihood estimate for the haplotype frequencies observed in cases ($O_1 \ldots O_k$), given the distribution in controls ($f_1 \ldots f_k$):

$$LOD_{ML} = \log_{10} \frac{P\left(O_1, \ldots, O_k \middle| \alpha e_j + (1-\alpha)(f_1, \ldots, f_k)\right)}{P\left(O_1, \ldots, O_k \middle| f_1, \ldots, f_k\right)}$$

where j is the haplotype on which the mutation arose, $1-\alpha$ is the recombination rate, and $e_j=1$ when $i=j$ and $e_j=0$ when $i \neq j$. The test produces maximum likelihood estimates for j and α.

So while a χ^2-based association finds any significant differences in haplotype frequencies, the HLR test models a specific scenario that is common in rapid selective events. The HLR test does not provide significantly more power than a single marker test in regions of high LD—in extreme cases, these regions may only have two haplotypes, and a multi-marker test will have the same power as a bi-allelic SNP test. But in regions where LD is low in controls and a single, long haplotype is prevalent in cases,

the HLR test is highly sensitive. The HLR test is a one-sided test and we ran separate tests for both drug resistance (called "risk") and drug sensitivity ("protect"). Results for drug sensitivity are available in Fig. S10, but are not reported generally as we are more interested in selective events for drug resistance.

We used PLINK to produce sliding window haplotypes across the genome and calculate haplotype frequencies for input to the HLR test. We produced input for all two, four and six-marker windows. The resulting LOD scores did not map well to known distributions, such as the χ^2 1-degree of freedom distribution. We instead converted the pointwise LOD scores to empirical pointwise *P*-values by performing approximately 370,000 permutations of the null model for each test condition. This allows us to calculate empirical *P*-values up to a significance of about $-\log_{10}(P\text{-value}) = 5.6$. Similar to the permuted Fisher's test, we preserved population-specific phenotype frequencies by only allowing permutations within each of our three defined populations. Resulting *P*-value distributions fit expectations well for the vast majority of test conditions (Fig. S9, S10) and the test demonstrates power to detect a number of loci at genome-wide significance (Fig. 2A, Table 1).

Figure M1. SNPs located in genes (28,576) were more likely to pass concordance filtering than intergenic SNPs (19,999).

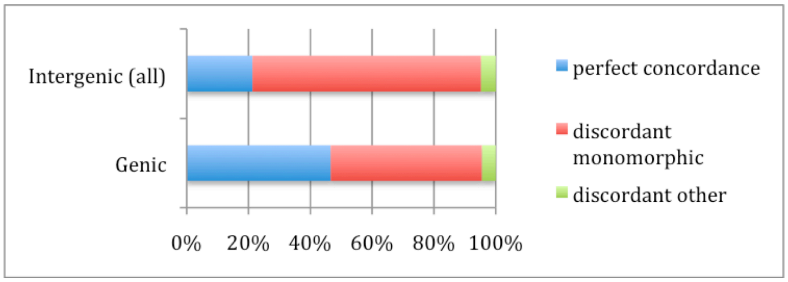

Figure M2. SNPs that were discovered from only one sequenced strain (35,727 SNPs) show a higher rate of monomorphism on the array than those with higher minor allele counts (12,848 SNPs). Any amount of this discordance may be explained by false discovery from sequencing data.

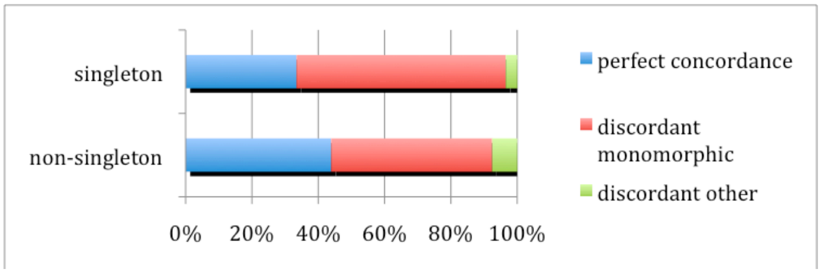

Figure M3. To show the effect of GC composition, we took 16bp of flanking sequence on each side of the SNP to construct a 3D7-based 33mer and calculated the percent GC. The window boundaries for the graph below are chosen as the octiles of the GC distribution. SNP performance appears to worsen at GC levels below 20%, which accounts for roughly half of the SNPs.

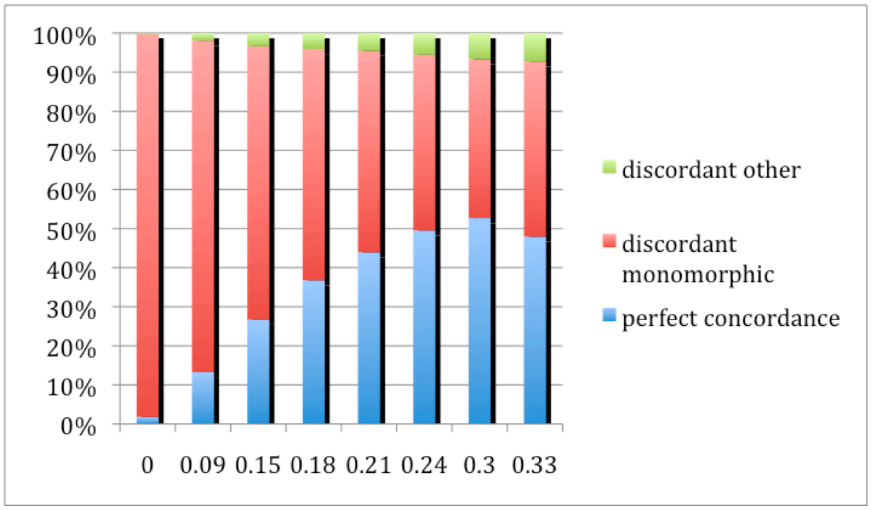

Figure M4. The effect of unique sequences in flanking regions. Although the initial design of the array excluded probes that had exact matches elsewhere in the genome, many of the remaining SNPs are in neighborhoods that contain 1 or 2 base mismatch similarity to other parts of the genome. We took 16bp of flanking sequence on each side of the SNP to construct a 3D7-based 33mer and aligned it to 3D7 using SSAHA (word length 10, step length 1, max gap 2, max insert 1, min hits 24) [15]. 28,352 SNPs aligned uniquely to their location of origin. The 20,223 SNPs that aligned in multiple locations showed a much higher rate of discordance.

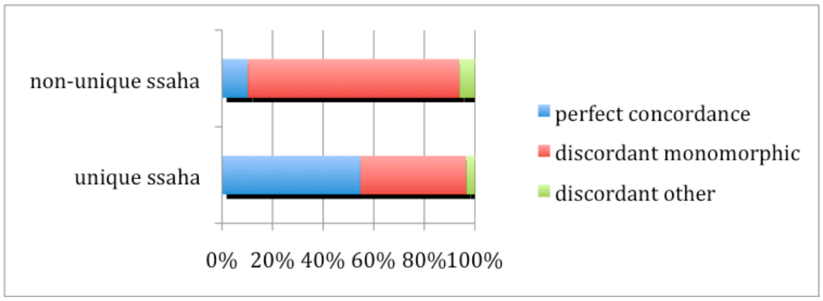

Figure M5. A histogram of marker spacing. Most markers are spaced closely with a few large gaps in coverage. The mean spacing of concordant markers is 1316bp with a median spacing of 444bp.

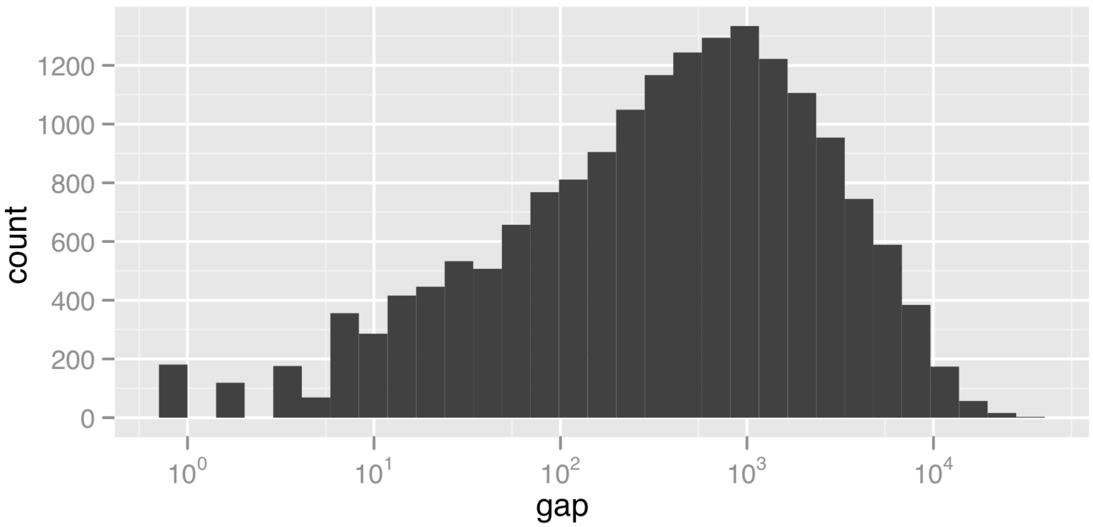

Table M1. Statistics on marker spacing (gaps) by chromosome. The table gives the number of gaps, as well as the median, mean gaps, 90% percentile (90% gap), and maximum gap length (max gap) by chromosome (1 – 14).

	number of gaps	median gap	mean gap	90% gap	max gap	sum of gaps
**all**	17,568	444	1,316	3,639	39,535	23,118,100
**chr 1**	644	150	957	2,968	18,798	616,104
**chr 2**	855	346	1,103	3,090	16,236	942,754
**chr 3**	825	485	1,282	3,300	24,076	1,057,642
**chr 4**	1,462	182	817	2,042	39,535	1,195,109
**chr 5**	977	447	1,344	3,865	17,583	1,312,630
**chr 6**	1,088	422	1,298	3,801	24,856	1,412,604
**chr 7**	1,599	225	935	2,446	19,341	1,495,349
**chr 8**	1,100	454	1,284	3,319	24,320	1,412,004
**chr 9**	1,087	557	1,415	3,820	27,661	1,538,216
**chr 10**	1,113	533	1,520	4,121	34,097	1,691,290
**chr 11**	1,357	636	1,497	4,115	21,921	2,031,460
**chr 12**	1,678	463	1,351	3,737	17,010	2,266,590
**chr 13**	1,831	669	1,562	4,333	19,966	2,860,436
**chr 14**	1,952	768	1,683	4,516	23,942	3,285,912

Figure M6. Final concordant marker density across all fourteen chromosomes.

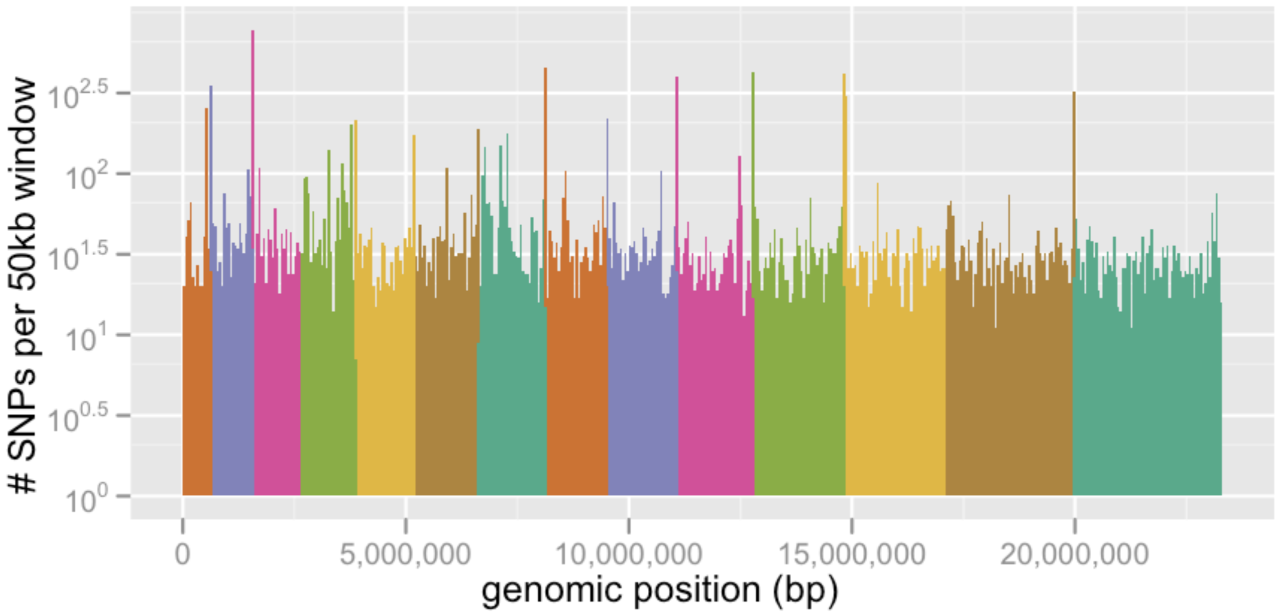

Figure M7. Distribution of markers per gene: 59% of all *P. falciparum* genes contain at least one concordant marker. Below is a plot of the number of markers per gene. Most genes that have markers have only one or two (median marker count per gene is two). Mean marker count per gene is 4.1.

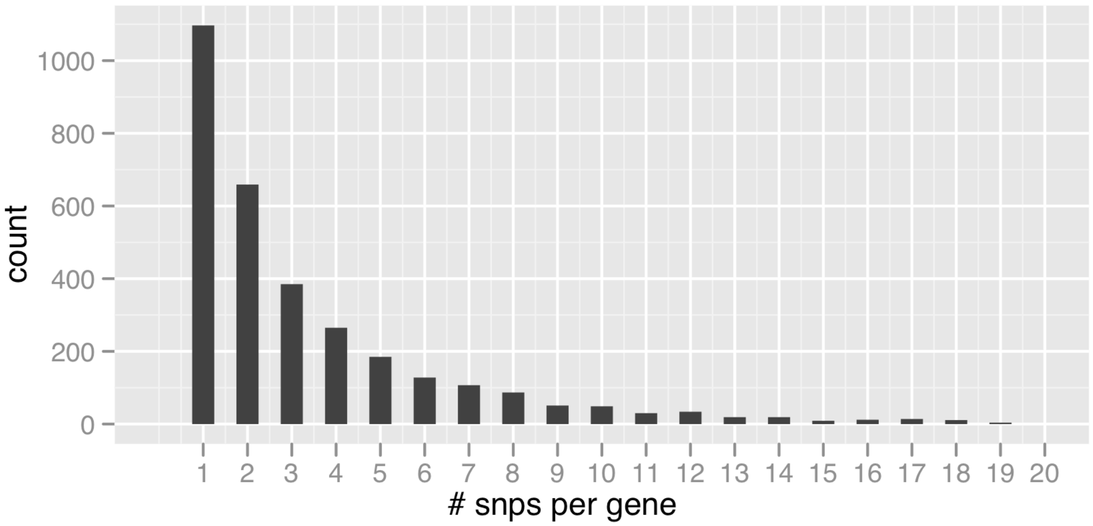

References

1. Duraisingh MT, Jones P, Sambou I, von Seidlein L, Pinder M, et al. (2000) The tyrosine-86 allele of the pfmdr1 gene of Plasmodium falciparum is associated with increased sensitivity to the anti-malarials mefloquine and artemisinin. Mol Biochem Parasitol 108: 13-23.
2. Volkman SK, Sabeti PC, DeCaprio D, Neafsey DE, Schaffner SF, et al. (2007) A genome-wide map of diversity in Plasmodium falciparum. Nat Genet 39: 113-119.
3. Kent WJ (2002) BLAT--the BLAST-like alignment tool. Genome Res 12: 656-664.
4. Altshuler D, Pollara VJ, Cowles CR, Van Etten WJ, Baldwin J, et al. (2000) An SNP map of the human genome generated by reduced representation shotgun sequencing. Nature 407: 513-516.
5. Jeffares DC, Pain A, Berry A, Cox AV, Stalker J, et al. (2007) Genome variation and evolution of the malaria parasite Plasmodium falciparum. Nat Genet 39: 120-125.
6. Ribacke U, Mok BW, Wirta V, Normark J, Lundeberg J, et al. (2007) Genome wide gene amplifications and deletions in Plasmodium falciparum. Mol Biochem Parasitol 155: 33-44.
7. Rabbee N, Speed TP (2006) A genotype calling algorithm for affymetrix SNP arrays. Bioinformatics 22: 7-12.
8. Daniels R, Volkman SK, Milner DA, Mahesh N, Neafsey DE, et al. (2008) A general SNP-based molecular barcode for Plasmodium falciparum identification and tracking. Malar J 7: 223.
9. Neafsey DE, Schaffner SF, Volkman SK, Park DJ, Montgomery P, et al. (2008) Genome-wide SNP genotyping highlights the role of natural selection in Plasmodium falciparum population divergence. Genome Bio. pp. R171.
10. Roberts DJ, Craig AG, Berendt AR, Pinches R, Nash G, et al. (1992) Rapid switching to multiple antigenic and adhesive phenotypes in malaria. Nature 357: 689-692.
11. Kidgell C, Volkman SK, Daily J, Borevitz JO, Plouffe D, et al. (2006) A systematic map of genetic variation in Plasmodium falciparum. PLoS Pathog 2: e57.
12. Purcell S, Neale B, Todd-Brown K, Thomas L, Ferreira MA, et al. (2007) PLINK: a tool set for whole-genome association and population-based linkage analyses. Am J Hum Genet 81: 559-575.
13. Kang HM, Zaitlen NA, Wade CM, Kirby A, Heckerman D, et al. (2008) Efficient control of population structure in model organism association mapping. Genetics 178: 1709-1723.
14. Lindblad-Toh K, Wade CM, Mikkelsen TS, Karlsson EK, Jaffe DB, et al. (2005) Genome sequence, comparative analysis and haplotype structure of the domestic dog. Nature 438: 803-819.
15. Ning Z, Cox AJ, Mullikin JC (2001) SSAHA: a fast search method for large DNA databases. Genome Res 11: 1725-1729.
16. Patterson N, Price AL, Reich D (2006) Population structure and eigenanalysis. PLoS Genet 2: e190.
