# Supplementary material for: Identification and Functional Validation of the Novel Antimalarial Resistance Locus PF10_0355 in Plasmodium falciparum
Source: PLoS Genet. 2011 Apr 21;7(4):e1001383. doi: 10.1371/journal.pgen.1001383 (PMC3080868; doi:10.1371/journal.pgen.1001383)
Supplement: Supplementary file 11 [file pgen.1001383.s014.doc]

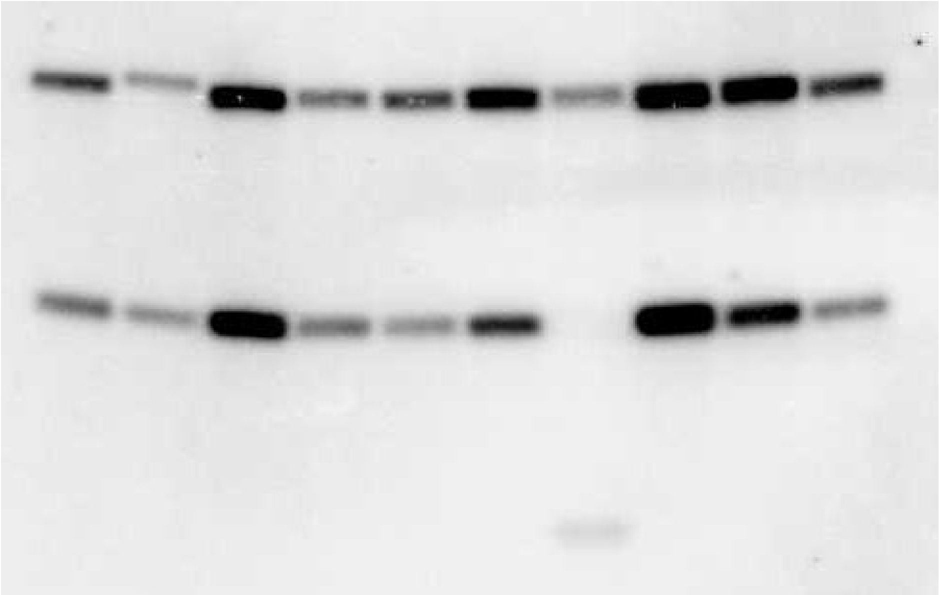

	V34.04	TM90C6A	TM90C2A	TM327	P26.04	P19.04	P11.02	GH2	D10	3D7
PF10_0355 copy number	1.54	**2.26**	**2.43**	**2.02**	1.21	1.18	0.53	**2.38**	1.06	1.00
HFN response	S	R	R	R	R	R	R	R	R	R
